# Resistance to medically important antimicrobials in broiler and layer farms in Cameroon and its relation with biosecurity and antimicrobial use

**DOI:** 10.3389/fmicb.2024.1517159

**Published:** 2025-01-15

**Authors:** Ronald Vougat Ngom, Andrea Laconi, Roberta Tolosi, Adonis M. M. Akoussa, Stephane D. Ziebe, Vincent M. Kouyabe, Alessandra Piccirillo

**Affiliations:** ^1^Department of Animal Production, School of Veterinary Medicine and Sciences, University of Ngaoundere, Ngaoundere, Cameroon; ^2^Department of Comparative Biomedicine and Food Science, University of Padua, Legnaro, Italy; ^3^National Veterinary Laboratory, Garoua, Cameroon

**Keywords:** antimicrobial resistance, antimicrobial use, resistance genes, poultry, Africa

## Abstract

**Introduction:**

Poultry production accounts for 42% of Cameroonian meat production. However, infectious diseases represent the main hindrance in this sector, resulting in overuse and misuse of antimicrobials that can contribute to the emergence and dissemination of antimicrobial resistance (AMR). This study aimed to evaluate the prevalence of antimicrobial resistance genes (ARGs) conferring resistance to carbapenems (*bla_VIM-2_* and *bla_NDM_*), (fluoro) quinolones (*qnrS*, *qnrA*, and *qnrB*), polymyxins (*mcr1* to *mcr5*), and macrolides (*ermA* and *ermB*) in the poultry farm environment. Additionally, the study examined the relationship between these ARGs and biosecurity implementation, as well as farmers’ knowledge, attitudes, and practices toward antimicrobial use (AMU) and AMR, including their perception of AMR risk.

**Materials and methods:**

Fecal, drinking water, and biofilm samples from drinking water pipelines were collected from 15 poultry farms and subsequently analyzed by real-time PCR and 16S rRNA NGS.

**Results:**

All samples tested positive for genes conferring resistance to (fluoro) quinolones, 97.8% to macrolides, 64.4% to polymyxins, and 11.1% to carbapenems. Of concern, more than half of the samples (64.4%) showed a multi-drug resistance (MDR) pattern (i.e., resistance to ≥3 antimicrobial classes). Drinking water and biofilm microbial communities significantly differed from the one of the fecal samples, both in term of diversity (*α*-diversity) and composition (*β*-diversity). Furthermore, opportunistic pathogens (i.e., Comamonadaceae and Sphingomonadaceae) were among the most abundant bacteria in drinking water and biofilm. The level of biosecurity implementation was intermediate, while the knowledge and attitude of poultry farmers toward AMU were insufficient and unsuitable, respectively. Good practices toward AMU were found to be correlated with a reduction in polymyxins and MDR.

**Discussion:**

This study provides valuable information on resistance to medically important antimicrobials in poultry production in Cameroon and highlights their potential impact on human and environmental health.

## 1 Introduction

The poultry industry represents a vital sector in many African countries (Ochieng et al., 2021). In Cameroon, the second highest poultry producer in West and Central Africa (Monamele et al., 2019) the sector accounts for 4% of Gross Domestic Product (GDP). Eggs and poultry represent 14% of the population protein intake (MINEPIA, 2019) and 42% of the total meat production [GIZ (Deutsche Gesellschaft fur Internationale Zusammenarbeit GmbH), 2018], respectively. However, similarly to other livestock sectors, infectious diseases represent the main hindrance in Cameroonian poultry production, leading to increased antimicrobial use (AMU) for disease prevention and control (Moffo et al., 2020). The poultry sector is the main user of antimicrobials in Cameroon (Mouiche et al., 2020). Factors such as population growth (Demography - Cameroon Statista, 2024) and rising consumer demand for poultry products, along with the transition from small-to large-scale intensive production systems (Klein et al., 2018), are driving the increased use of antimicrobials.

In poultry farming, drinking water represents the most common route for administering antimicrobial drugs (AMDs). However, drinking water and its distribution systems can be contaminated with microbiological constituents (e.g., biofilm), affecting the stability and availability of AMDs. Biofilms can capture antimicrobials, contributing to treatment failures and the spread of antimicrobial resistance (AMR) (Sparks, 2009). To complicate the scenario, the overuse and misuse of antimicrobials, including medically important antimicrobials for human medicine, in Cameroonian poultry farms, can contribute to the emergence and dissemination of AMR (Moffo et al., 2022; Vougat Ngom et al., 2024a).

AMR poses a serious public health concern all over the world (FDA, 2020), with a higher impact recorded in Africa (Sartorius et al., 2024). Resistant genes and bacteria from livestock can reach humans through direct contact (e.g., farmers), the food chain, or drainage into water basins and water supplies (Berendonk et al., 2015; Hruby et al., 2016; Alban et al., 2017; Collineau et al., 2020). Biosecurity and farmers’ education are key strategies to reduce AMU, by mitigating the risk of introduction and spread of infectious diseases and by increasing their awareness on AMDs use and AMR, respectively (Postma et al., 2017; Rodrigues Da Costa et al., 2019). Improved biosecurity can also enhance the technical performances of reared flocks (Rodrigues Da Costa et al., 2019). Many phenotypic studies in Cameroon (Djuikoue et al., 2023; Leinyuy et al., 2023) have demonstrated widespread AMR in poultry farms. However, a recent review reported a lack of molecular studies to identify resistance genes (ARGs) to medically important antimicrobials from livestock (Vougat Ngom et al., 2024a). Furthermore, only one previous study investigated the knowledge the risk perception on AMR of Cameroonian poultry farmers (Moffo et al., 2020). This gap may be due to insufficient infrastructure for molecular analyses, yet this approach is highly recommended to improve the understanding of AMR dissemination in primary production.

In this context, the present study aimed to evaluate for the first time the presence of resistance determinants to medically important antimicrobials for human medicine in feces, drinking water, and biofilm from Cameroonian poultry farms. Additionally, it investigated the relationship between the ARGs and biosecurity implementation, as well as farmers’ knowledge, attitudes, and practices regarding AMU and their knowledge and perception of AMR risk.

## 2 Materials and methods

### 2.1 Study area

**Figure 1:**
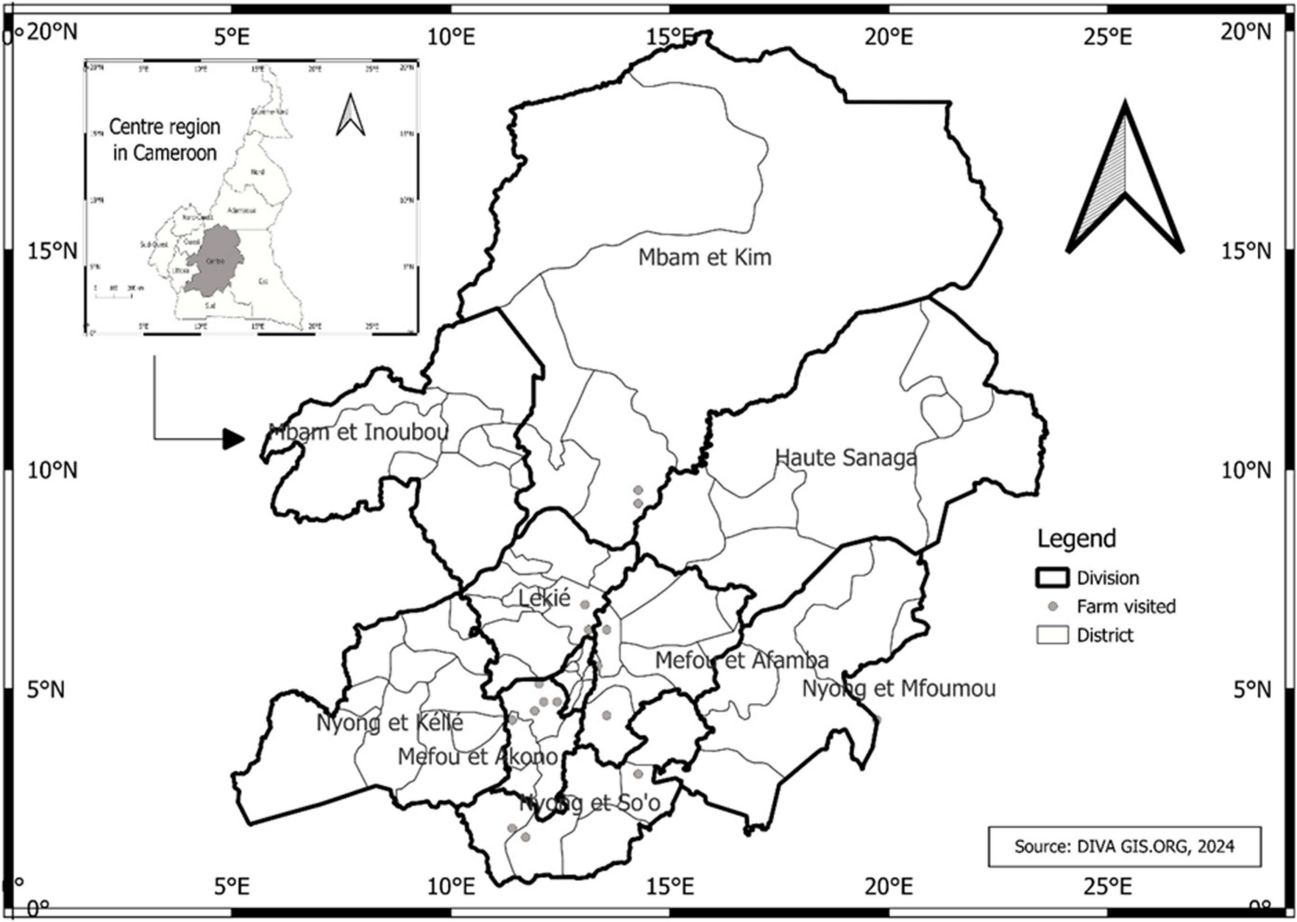
Map reporting the locations of farms in the Center Region of Cameroon. The poultry farms included in this study were in 6 (Méfou-et-Afamba, Méfou-et-Akono, Lekié, Mfoundi, Nyong-et-Mfoumou and Mbam-et-Kim) out of 10 divisions of the Center Region of Cameroon.

This study was conducted in the Center region of Cameroon (3° 52′- 6°14’ LN and 11°31′- 12°93′ LE) in August 2023 (Figure 1). The Center region was selected due to its significant poultry production, accounting for 15.8% of the national poultry population, following the West (23.2%) and North West (18.7%) regions. It also has the highest rate of chicken meat and egg consumption in the country (MINEPIA, 2019). The study was carried out in 6 of the 10 divisions in this region: Méfou-et-Afamba, Méfou-et-Akono, Lekié, Mfoundi, Nyong-et-Mfoumou and Mbam-et-Kim. Authorization for the study was obtained from the regional delegation in charge of livestock (N 00120/L/MINEPIA.SG/DREPIA-CE).

### 2.2 Study design and data collection

A cross-sectional survey was conducted in poultry farms using drinking water distribution systems (i.e., nipple drinkers, automatic bell water drinker, and pendular drinking system). Based on lists of poultry farmers from the division Delegations of the Ministry of Livestock, Fisheries and Animal Industries (MINEPIA), 18 poultry farmers were identified, and 15 agreed to participate. The purpose of the study was explained to the farmers before data collection. The demographics of participants are summarized in Table 1, indicating that the majority were male (66.7%), over 39 years of age (53.4%), had at least a secondary education level (66.7%), and had more than 5 years of experience in poultry farming (60.0%). Most farms were constructed before 2020 (73.3%) using the farmers’ own funds (80.0%) (Table 2). The number of chickens reared varied from 1,000 to 20,000 per cycle, with most farms (46.7%) housing between 2,000 and 5,000 birds, and having at least three barns (60.0%). Eight farms reared layers, six broilers and one both. Broiler farms reared at least five batches per year. Day-old chicks were purchased from feed (antibiotic-free) or chick sellers (53.3%) or directly from hatcheries (46.7%). Feeds were generally farm made and distributed manually. Farms sourced water from traditional (53.3%) and modern (40.0%) wells, or both (6.6%).

**Table 1:** Demographics of poultry farmers participating in the study.

Variable	Number (*n*)	Percentage (%)
Gender		
Male	10	66.7
Female	5	33.3
Educational level		
Primary	1	6.6
Secondary	7	46.7
Higher	7	46.7
Age (years)		
30–39	1	6.6
40–49	6	40
50–59	5	33.3
60–69	3	20
Training in poultry farming		
Yes	7	46.7
No	8	53.3
Poultry farming as main activity		
Yes	13	86.7
No	2	13.3
Year of experience in poultry farming		
2–5	6	40
6–9	2	13.3
10–12	3	20
≥ 13	4	26.7

**Table 2:** Characteristics of poultry farms included in the study.

Variable	Number (*n*)	Percentage (%)
Received fundings for farming		
Yes	3	20
No	12	80
Type of poultry		
Broilers	6	40
Layers	8	53.3
Broilers and layers	1	6.6
Year of construction of the farm		
10	1	6.6
7–9	3	20
6–3	7	46.7
< 3	4	26.7
Number of animals present at the farm		
1,000	1	6.6
2,000-5,000	7	46.7
6,000-10,000	3	20
> 10,000	4	26.7
Number of barns per farm		
≤2	9	60
3–4	3	20
6	2	13.3
34	1	6.6
Number of broilers batches per year		
5	2	33.3
6	3	50
7	1	16.7
Density in the farm		
<6	9	60
6–9	5	33.4
>9	1	6.6
Source of one-day chicks		
Feed or chicks dealers	8	53.3
Hatchery	7	46.7
Origin of feed		
Farm made	12	80
Produced by a company	3	20
Distribution of feed		
Manual	12	80
Automatic	3	20
Water source		
Modern well	6	40
Traditional well	8	53.3
Both	1	6.6

A questionnaire (Supplementary material 1) adapted from Moffo et al. (2020, 2022) and Chowdhury et al. (2023) was used for data collection. The questionnaire, pre-tested in five farms (not included in the paper), contained different sections related to farm characteristics, biosecurity practices, farmers’ knowledge, attitudes and practices on AMU, and knowledge and risk perception on AMR. Biosecurity (i.e., internal and external) implementation was assessed using a risk-based scoring tool (Moffo et al., 2020). Data collection involved face-to-face interviews with the farm owners or managers using a hard copy of the questionnaire, complemented by direct observations. Interviews targeted workers involved in the decision process of antimicrobial administration and were conducted by two trained final-year veterinary students under the supervision of a full researcher. Each farm visit lasted about 2 hours. Questionnaires are available upon request to the first author.

### 2.3 Sampling procedure

Fecal, drinking water, and biofilm samples were collected in each farm. Samples were taken from all barns if one or two were present, and from two randomly selected barns if more than two were present. At least 25 mg of fecal droppings were collected from 10 locations using sterile spatulas. Biofilm samples were collected by swabbing the inside of 10 randomly selected water lines using sterile cotton swabs, which were placed in a separate bottle after removing the wooden shaft. Swabs collected from the same farm were pooled together before DNA extraction. Two liters of drinking water were collected at the end of all water lines using sterile bottles. Samples were labeled, stored on ice, and either processed immediately (<12 h) or stored at −80°C until analyzed. To minimize disease transmission risk, a maximum of two farms per day were sampled, with strict biosecurity measures observed before (e.g., take an appointment, no visit to other farms on the same day), during (e.g., cleaning and disinfection, parking away from the farms, wear farm-specific clothing and shoes,) and after visiting each farm (e.g., cleaning and disinfection, no visit to other farms on the same day).

### 2.4 DNA extraction

Water samples were filtered using a vacuum pump and 0.45 μm filter membranes (Sartorius Stedim Biotech GmbH, Germany). Filters were cut into small pieces and placed in ZR BashingBeadTM Lysis Tubes with 750 μL of ZymoBIOMICS lysis solution. For fecal samples, 200 mg were directly placed in lysis tubes. Swabs were placed in ZR BashingBead™ Lysis Tube with 750 μL of ZymoBIOMICS™ lysis solution and vortexed for 30 min before removing the swabs. DNA was extracted using the DNA Miniprep Kit (ZymoBIOMICS™) according to the manufacturer’s instructions at the National Veterinary Laboratory (LANAVET) annex of Yaoundé, Cameroon. DNA extracts were shipped at controlled temperature to the Department of Comparative Biomedicine and Food Science of the University of Padua (Italy) for further analyses. DNA quantity was assessed using the Qubit dsDNA High Sensitivity kit (Thermo fisher Scientific) (Supplementary material 2). On a subset of samples (*n* = 10) representative of all sample-type and range of DNA concentration (i.e., low, medium, high), DNA quality was assessed using the Agilent 2,100 Bioanalyzer (Agilent Technologies, Palo Alto, CA, USA).

### 2.5 16S rRNA gene amplification, sequencing, and data analysis

Microbial communities were investigated by amplifying the V3-V4 regions of the 16S rRNA gene and sequencing using next-generation sequencing (NGS). Libraries were prepared as described by Laconi et al. (2022a) and sequenced using the Illumina MiSeq sequencing platform (San Diego, California, USA) with a 2 × 300 bp paired-end approach. Data analysis and taxonomic assignment of microbial communities were performed using the DADA2 package (Callahan et al., 2016; Bolyen et al., 2019) and the SILVA-Naive Bayes sklearn trained database (Yilmaz et al., 2014), respectively, within the Quantitative Insights into Microbial Ecology 2 (QIIME2 version 2023.5). After data filtering, total sum normalization (TSS), and SquareRoot transformation, microbial communities were assessed using MicrobiomeAnalyst^1^. In detail, the microbial diversity within each group (*α*-diversity) was assessed using Shannon’s and Simpson’s indexes, while the Permutational Multivariable Analysis of Variance (PERMANOVA) based on the Bray-Curtis dissimilar measure was used to assess differences among groups (*β*-diversity). *β*-diversity was visualized using Principal Coordinate Analysis (PCoA) and Non-metric Multidimensional Scaling (NMDS), while microbial communities’ composition was investigated using heatmaps (pheatmap v1.0.12 package within Rstudio v2024.04.2). Linear discriminant analysis (LDA) effect size (LEfSe) was used to identify taxa associated with different sample matrices. Raw sequence reads are deposited in the NCBI Short Read Archive under the accession number PRJNA1123609. After the quality-filter step, removal of chimeric fragments and reads merging, a total of 396,859 reads were obtained, with 12,501 different features and an average of 8,819 sequences per individual sample.

### 2.6 Antimicrobial resistance genes (ARGs) detection by real-time PCR

All samples were tested for 12 ARGs, conferring resistance to carbapenems (*bla_VIM-2_* and *bla_NDM_*), macrolides (*ermA* and *ermB*), (fluoro) quinolones (*oqxA*, *oqxB*, and *qnrS*), and polymyxins (*mcr-1*, *mcr-2*, *mcr-3*, *mcr-4*, and *mcr-5*), using real-time PCR with PowerUp™ SYBR® Green Master Mix (Thermo Fisher Scientific) in a LightCycler®480 Roche (Roche, Basel, Switzerland) as previously described (Laconi et al., 2022b). The assays, primers pairs, melting temperatures, and positive controls are available in Supplementary material 3.

### 2.7 Statistical analysis

Binomial analysis assessed differences in ARGs occurrence (binary outcome variable), class-level resistance (i.e., at least one ARG per antimicrobial class) and MDR (i.e., resistance to at least three antimicrobial classes) across sample types, farms, water source types, water treatments, and AMU using Fisher’s exact test. To assess the association between the relative abundance of microbial taxa at family level and the ARGs detected at a minimum prevalence level of 10% in all samples, multivariate regression analysis was used to jointly regress the dependent variables (i.e., log-transformed relative abundances taxa) on the same independent variables (i.e., presence/absence of ARGs). Multiple logistic regression analysis was used to test for significance differences in ARGs prevalence, class-level resistance, and MDR across all the aforementioned explanatory variables, along with biosecurity scores and farmers’ knowledge, practices, AMU and AMR awareness, and risk perception regarding AMR. Descriptive statistics summarized data concerning farmers’ knowledge and risk perception on AMU and AMR. Knowledge, attitude and practices data were analyzed as described by Moffo et al. (2020). Briefly, answers of the interviewees were coded into binary outcomes, with 1 representing sufficient knowledge, desirable attitude, appropriate practice toward AMU/AMR and risk perception of AMR, and 0 represented insufficient knowledge, unsuitable attitude, inappropriate practice, and risk perception. The sum of responses recorded as binary outcome for each farmer for each individual category was divided by the total number of items in each category to obtain the average score for each (Caudell et al., 2019). Biosecurity scores were categorized using the following 3-level scoring system; “low” for scores ≤40%; “intermediate” 40% < scores <80%; and “high” scores ≥80%. Statistical analysis and data visualization were carried out in GraphPad Prism (version 10.2.3)^2^.

## 3 Results

### 3.1 Bacterial communities’ composition and diversity

**Figure 2:**
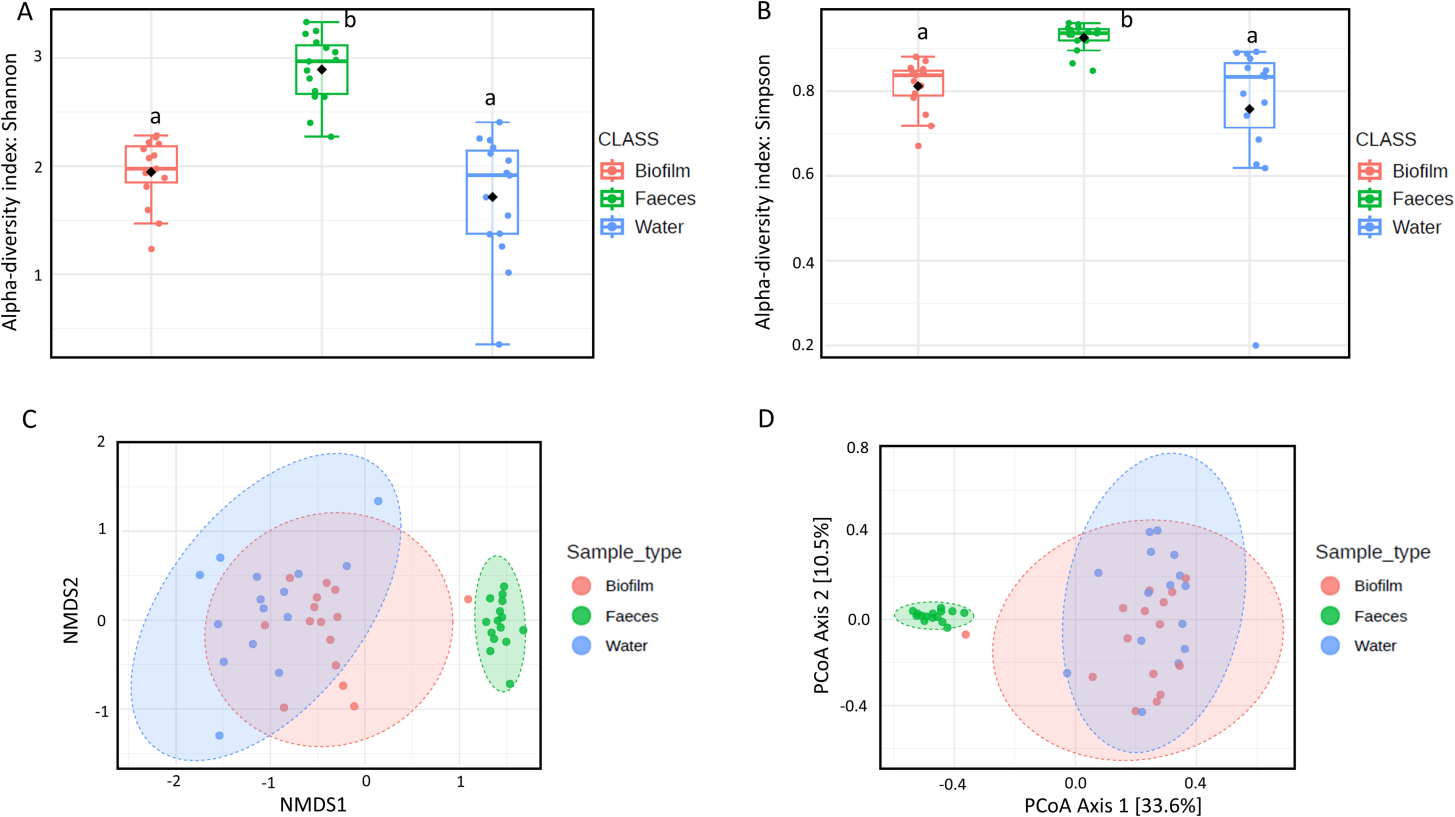
*α*-Diversity within each sample using **(A)** Shannon’s and **(B)** Simpson’s indexes. Boxplots represent 25th to 75th percentiles; different letters indicate significant differences within the α-diversity indexes (*p* < 0.05). *β*-Diversity between sample types according to Bray-Curtis distances using **(C)** Non-metric Multidimensional Scaling (NMDS) and **(D)** Principal Coordinate Analysis (PCoA).

**Figure 3:**
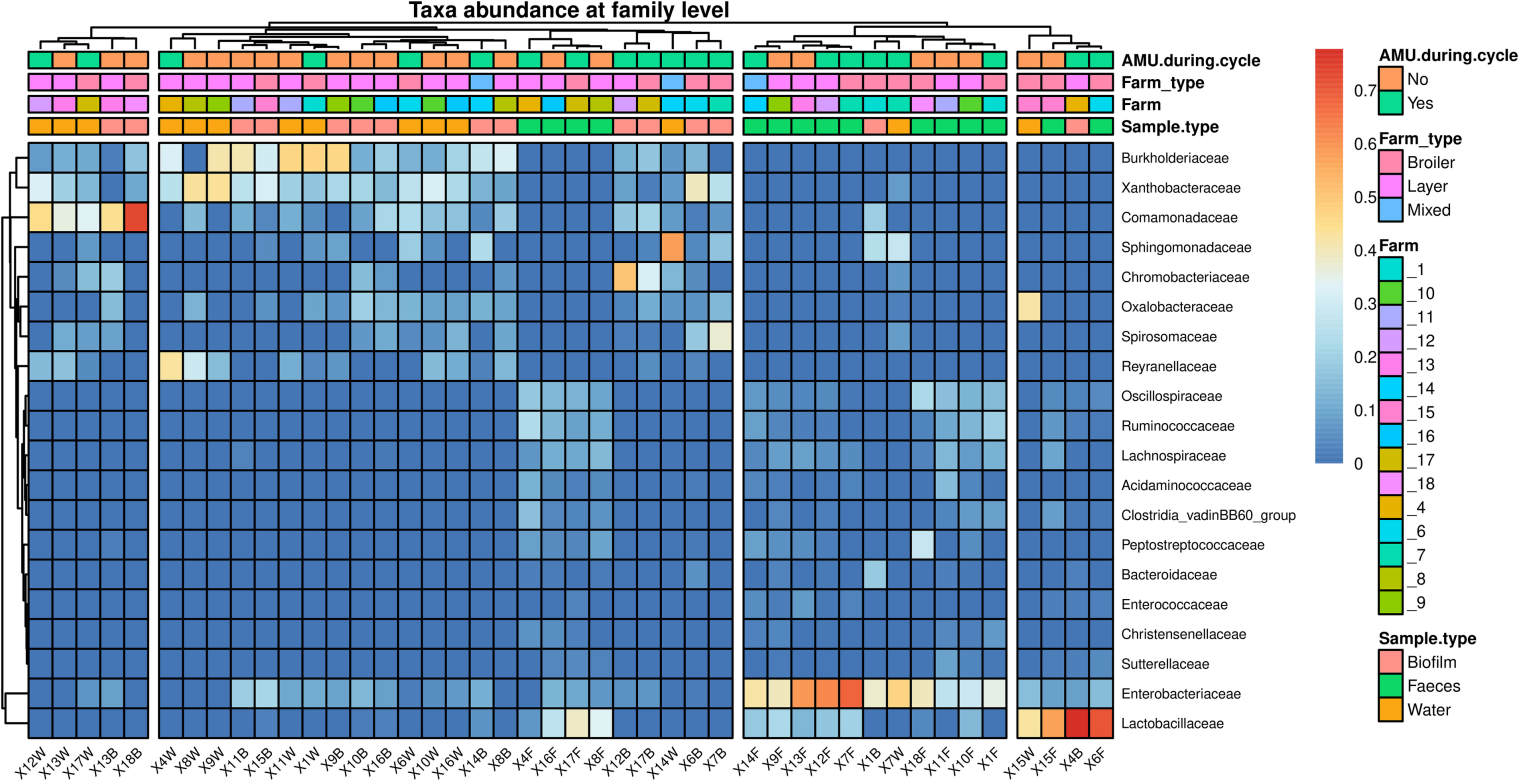
Heatmap representing the microbial community composition of fecal, water, and biofilm samples at family level. Each row represents a different taxon, while each column represents an individual sample. Samples are clustered according to the abundance of taxa. Taxa are displayed in a color gradient from red to blue, representing high and low abundance, respectively.

The microbial communities’ composition of fecal, drinking water and biofilm samples was explored using 16S rRNA gene sequencing. The *α*-diversity, assessed at operational taxonomic unit (OTU) level and expressed using Shannon’s and Simpson’s indexes, was significantly higher in feces compared to drinking water (*p* = 0.0005) and biofilm (*p* = 0.0024), (Figures 2A,B). PERMANOVA analysis indicated significant differences in microbial communities among the three matrices (*p* = 0.0010). However, NMDS and PCoA graphs (Figures 2C,D) showed that biofilm and drinking water microbiota were less distant from each other (*p* = 0.0170) compared to fecal samples (*p* = 0.0010). No significant differences in α-diversity and *β*-diversity were observed within the sample type between farms that administered antimicrobials to birds in the 2 weeks preceding the sampling (AMU_Farms) and those that did not (No_AMU_Farms) (Supplementary material 4). The heatmap at the family level (Figure 3) clustered samples primarily by sample type rather than antimicrobials used. Indeed, two main clusters could be observed, one including the majority of fecal samples and one including mainly biofilm and drinking water samples, interspersed with one another. Lactobacillaceae and Entorobacteriaceae dominated fecal samples, while Burkholderiaceae, Xanthobacteraceae, and Comamondaceae were dominant in drinking water and biofilm. Indeed, among the six taxa significantly more abundant in fecal samples, LEfSe analysis identified Enterobacteriaceae (linear discriminant analysis (LDA) = 6.01) and Lactobacillaceae (LDA = 5.96) (Supplementary material 5). Other notable taxa associated with the fecal microbiota were Oscillospiraceae (LDA = 5.79), Ruminococcaceae (LDA = 5.60), Spiromaceae (LDA = 5.45), and Peptostreptococcaceae (LDA = 5.39). Xanthobacteraceae (LDA = 6.05), Reyranellaceae (LDA = 5.95), Comamonadaceae (LDA = 5.83), and Sphinomonadaceae (LDA = 5.64) were more abundant in drinking water, while Burkholderiaceae (LDA = 5.93), Chromobacteriaceae (LDA = 5.69), Weeksellaceae (LDA = 5.63), Lachnospiraceae (LDA = 5.45), and Oxalobacteraceae (LDA = 5.37) in biofilm.

### 3.2 ARGs prevalence

**Figure 4:**
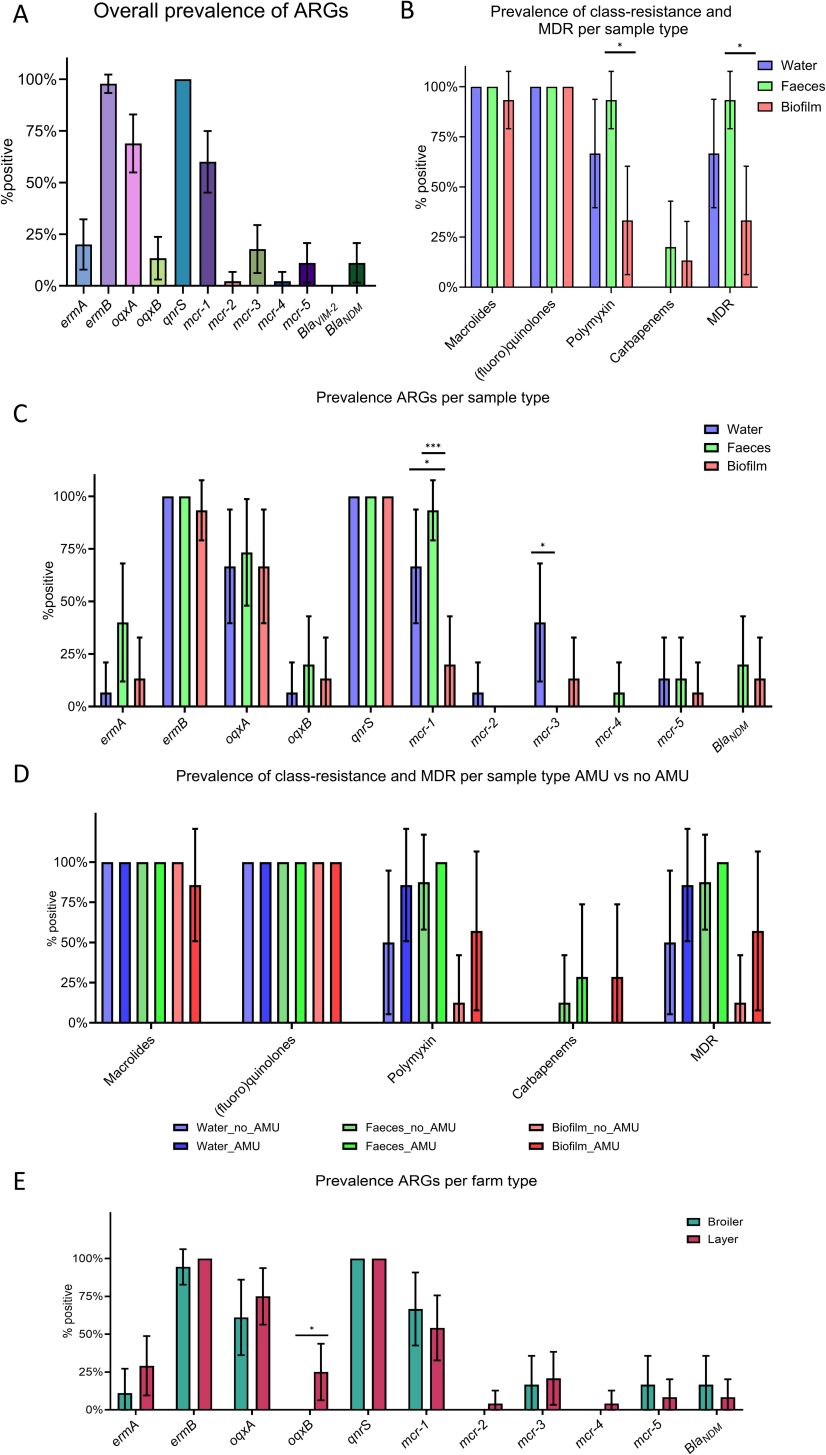
Prevalence of target genes in fecal, water, and biofilm samples. **(A)** Overall prevalence of antimicrobial resistance genes (ARGs). **(B)** Prevalence of class-resistance and multi-drug resistance (MDR) according to sample type. **(C)** Prevalence of ARGs according to sample type. **(D)** Prevalence of class-resistance and MDR according to sample type and antimicrobial use. **(E)** Prevalence of ARGs according to farm type (i.e., broilers and layers). *p* < 0.05 shown as * and *p* < 0.001 as ***. *p*-values referred to the binomial analysis. Whiskers represent 95% CI.

The presence of ARGs in fecal, drinking water and biofilm samples was assessed by real-time PCR. 11 out of 12 ARGs were detected in at least one sample, with *bla_VIM2_*, encoding resistance to carbapenems, being the only one undetected. All samples tested positive for *qnrS*, while *ermB* (97.8, 95% confidence interval (CI) 93.3–100%) was the second most prevalent ARG, followed by *oqxA* (68.9, 95% CI 54.8–82.9%), *mcr-1* (60.0, 95%CI 45.1–74.9%), *ermA* (20.0, 95% CI 6.0–40.4%), *mcr-3* (17.8, 95% CI 6.2–29.4%), *oqxB* (13.3, 95% CI 3.0–23.7%), *mcr-5* and *bla_NDM_* (11.1%; 95% CI 1.6–20.7%), and *mcr-2* and *mcr-4* (2.2, 95% CI 0–6.7%) (Figure 4A). When considering antimicrobial classes, all samples tested positive for at least one gene conferring resistance to (fluoro) quinolones, 97.8% (95% CI 93.3–100%) to macrolides, 64.4% (95% CI 49.9–79.0%) to polymyxins, and 11.1% (95% CI 1.6–20.7%) to carbapenems. Additionally, 64.4% (95% CI 49.9–79.0%) of the samples showed resistance to at least three antimicrobial classes (MDR).

Although ARGs, class-level resistance, and MDR showed a similar distribution in the different matrices, some differences were observed (Figures 4B,C). Binomial analysis indicated that *mcr-1* (*p* = 0.0001), polymyxins resistance genes (i.e., *mcr-1* to *mcr-5*) (*p* = 0.0017), and MDR (*p* = 0.0017) were more prevalent in fecal samples compared to biofilm. Logistic regression analysis confirmed that biofilm samples were less likely to harbor *mcr-1* (*p* = 0.0011, odd ratio (OR) = 0.0012, 95% CI 0.0001–0.0552), genes conferring resistance to polymyxins (*p* = 0.0025, OR = 0.0021, 95% CI 0.0003–0.0552), and MDR (*p* = 0.0024, OR = 0.0021, 95% CI 0.0002–0.05519) compared to feces. Binomial analysis showed that *mcr-1* was also significantly less prevalent in drinking water (66.7, 95% CI 39.6–93.7%, *p* = 0.0253) compared to fecal samples. However, multiple logistic regression did not confirm this difference (OR = 0.0558, 95% CI 0.0139–0.7018, *p* = 0.0537). Furthermore, mcr-3 was detected only in drinking water and biofilm, *bla_NDM_* only in feces and biofilm, *mcr-2* only in drinking water, and *mcr-4* only in fecal samples.

Differences between farm type (i.e., broiler and layer farms) was also investigated and the binomial analysis identified a positive association between layer farms and the *oqxB* gene, encoding resistance to (fluoro) quinolones (Figure 4E). The analysis investigating the effect of antimicrobial treatments 14 days prior to sampling did not show any statistical difference in the prevalence of ARGs, class-level resistance, nor MDR between AMU_Farms and No_AMU_Farms (Figure 4D and Supplementary material 6). However, within each matrix, a trend of increasing prevalence of polymyxins and MDR was observed in farms where antimicrobials were recently used, and *bla_NDM_* was detected in biofilms from the AMU_Farms group only. Farms using different water sources (i.e., drilling or well water) and/or water treatments showed similar distributions of ARGs, class-level, and MDR (*p* > 0.05).

### 3.3 Association between microbial communities and ARGs

Multivariate regression analysis explored possible associations between the most abundant taxa at family level and the presence/absence of ARGs. Among the nine ARGs included (i.e., prevalence >10% of samples), only *mcr-1* was positively associated with Xanthobacteraceae (*p* = 0.0141, O.D. = 4.467, 95% CI 1.62–19.46).

### 3.4 Farmers’ knowledge, practices and attitude toward AMU

**Figure 5:**
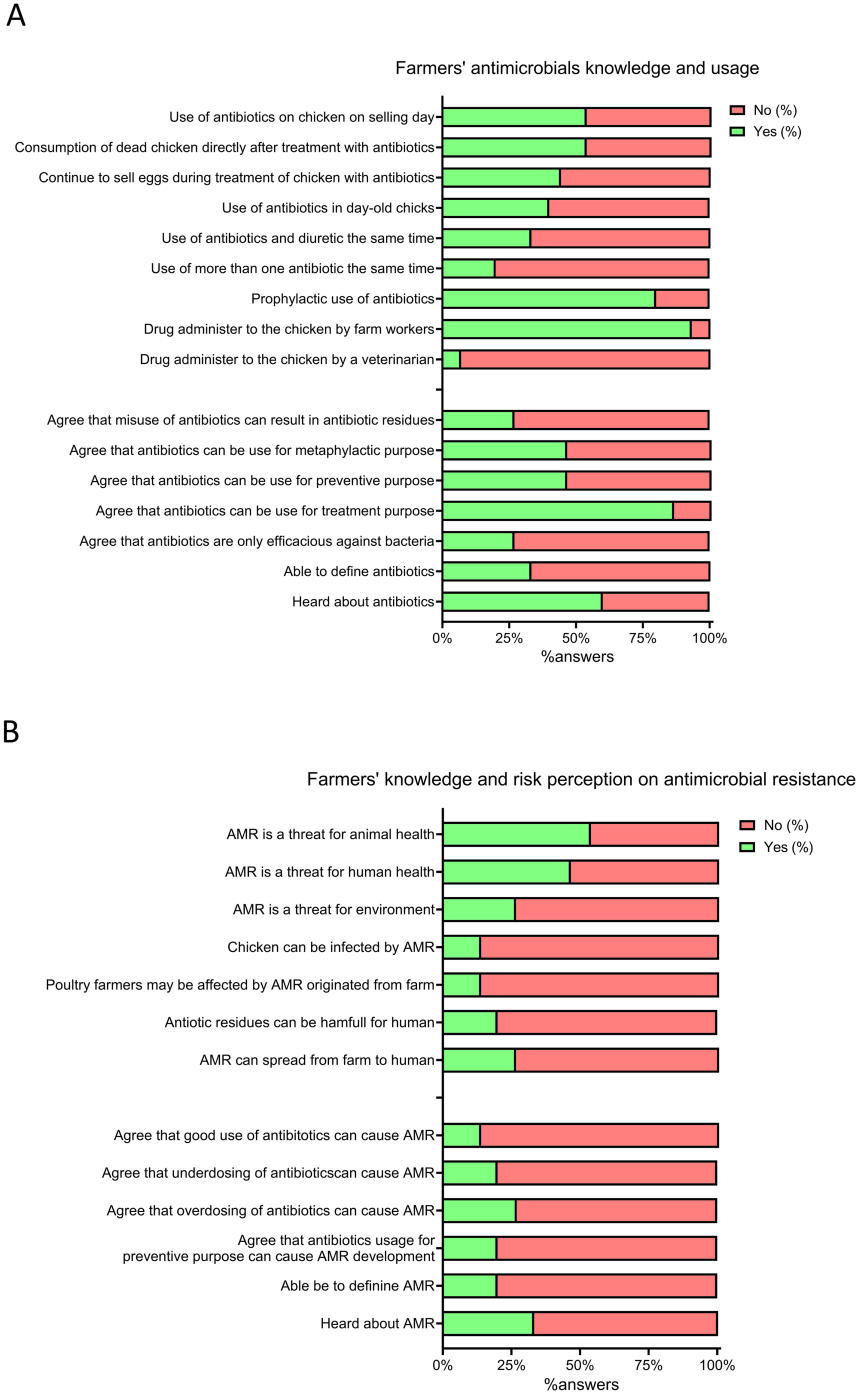
Farmer’s knowledge, practices and perception about antimicrobial use (AMU) and antimicrobial resistance (AMR). **(A)** Farmer’s antimicrobial knowledge and usage. **(B)** Farmer’s knowledge and risk perception toward antimicrobial resistance. Positive answers (%) are reported in green, while negative answers (%) are reported in red.

The average knowledge and attitude scores on AMU of poultry farmers were insufficient (0.41 ± 0.14) and unsuitable (0.43 ± 0.11), respectively, while the average score on AMU practices was appropriate (0.74 ± 0.07). Sixty percent of participants had heard about antimicrobials, but only 33.3% could provide a proper definition (Figure 5A). Only 26.7% of respondents agreed that antimicrobial misuse could lead to residues in poultry meat. Most farms (93.3%) had farm workers administering antimicrobials, and 80% of farmers used antibiotics for preventive purposes. More than half (53.3%) administered antimicrobials on the selling day and consumed dead chicken shortly after treatment. Almost half of the layer farmers (44.4%) sold eggs while birds were under antimicrobial treatment (Figure 5A).

Table 3 reports the AMDs used in the visited farms. Less than half of farmers (40.0%) bought AMDs based on a veterinarian prescription. Notably, among the most commonly used antimicrobials, 50% are listed as medically important antimicrobials and are also used for prophylaxis treatments. Enrofloxacin and colistin, used by 33.3 and 20.0% of farmers respectively, are listed as highest priority critically important antimicrobials (HPCIA). The respondents declared that, except for colistin, none of these antimicrobials seemed to be effective for the treatment of bacterial infections.

**Table 3:** Antimicrobials used in the poultry farms involved in the study.

Variable	Number (*n*)	Percentage (%)
How do you choose the drug to buy?		
Based on a veterinary prescription	6	40
Other (price, availability, feed seller recommendation)	6	40
Known efficacy	3	20
Do you follow the duration of the treatment?		
Yes	10	66.7
No	5	33.3
Do you think drugs can expire?		
Yes	9	60
No	6	40
Antibiotics mostly used		
Oxytetracycline	4	26.7
Colistin	3	20
Tylosin	1	6.7
Norfloxacin	2	13.3
Doxycycline	3	20
Amoxicillin	2	13.3
Enrofloxacin	5	33.3
Lincomycin	2	13.3
Antibiotics used for preventive purpose		
Colistin	1	6.7
Tylosin	2	13.3
Oxytetracycline	1	6.7
Sulfamide	2	13.3
Enrofloxacin	1	6.7
Doxycycline	1	6.7
Norfloxacin	1	6.7
Antibiotics which seems to be no longer efficacious		
Oxytetracycline	4	26.7
Doxycycline	2	13.3
Norfloxacin	2	13.3
Enrofloxacin	5	33.3
Amoxicillin	2	13.3
Tylosin	2	13.3
Antibiotics used during the 2 weeks prior samples collection		
Colistin	2	13.3
Tylosin	1	6.7
Oxytetracycline	1	6.7
Enrofloxacin	1	6.7
Doxycycline	1	6.7
Norfloxacin	1	6.7
Amoxycillin	1	6.7
Ciprofloxacin	1	6.7
None	8	53.3

### 3.5 Farmer’s knowledge and risk perception of AMR

The mean AMR knowledge and risk perception scores of poultry farmers were insufficient (0.22 ± 0.20) and inappropriate (0.34 ± 0.12), respectively. Only 33% of farmers were aware of AMR; indeed, less than a quarter of respondents (13.3%) recognized that antimicrobial misuse could lead to AMR development and that AMR could be harmful to themselves and their birds (Figure 5B). A minority acknowledged AMR as a threat to humans (46.7%) and the environment (26.7%), and only 26.7% mentioned AMR could spread from the farm environment to humans.

### 3.6 Level of implementation of biosecurity measures in poultry farms

Overall, the level of biosecurity implementation was intermediate (55.2 ± 10.5%), with higher internal (60.3 ± 9.1%) than external (50.1 ± 9.7%) biosecurity. Layer farms (61.9 ± 8.1%) were more compliant than broiler farms (45.2 ± 8.5%). Microbiological and physico-chemical controls of drinking water (e.g., pH, alkalinity, aerobic mesophilic counts) were routinely performed only in 13.3% of visited farms.

### 3.7 Association between biosecurity, AMU and AMR

Associations between ARGs prevalence, class-level resistance, and MDR and several correlates were investigated using multiple logistic regression analysis. No association between the scores of internal and external biosecurity and any of the independent variables considered (i.e., ARGs, class-level resistance, and MDR) were observed. Similarly, farmers’ knowledge on AMR and risk perception did not correlate with ARGs, class-level resistance, or MDR. However, when considering farmers’ attitude, knowledge, and practices on AMU, the latter was found to be associated with decreased resistance to polymyxin (*p* = 0.0401, OR = 0.8084, 95% CI 0.6223–0.9606) and MDR (*p* = 0.0374 and OR = 0.7576, 95% CI 0.5519–0.9148).

## 4 Discussion

### 4.1 Microbial communities differ among feces, drinking water and biofilm

The microbial community composition of poultry fecal samples aligns with that described in previous studies (Laconi et al., 2022a; Jensen et al., 2023; Meng et al., 2023), where Lactobacillaceae and Enterobacteriaceae were dominant. Similarly, the dominant taxa in drinking water and biofilm samples are consistent with those detected in drinking water distribution systems for human and livestock consumption (Kelly et al., 2014; Van Assche et al., 2019; Zhu et al., 2021). Notably, bacteria belonging to the most abundant families in drinking water and biofilm (i.e., Comamonadaceae and Sphingomonadaceae) can act as opportunistic pathogens in poultry carrying multiple resistance determinants (Willems, 2014; Ma et al., 2017). This finding suggests that more effort should be put into proper application of cleaning and disinfection procedures of the water pipelines, such as performing chlorine treatments between cycle, to reduce the abundance of these bacteria (Meng et al., 2023). While microbial communities of drinking water and biofilm seem to partially overlap, significant differences in richness and composition between these communities and the fecal microbiota were observed. In accordance with previous studies (Piccirillo et al., 2024), the latter showed higher richness and diversity (*α*-diversity) compared to the former. These differences in microbiota composition and diversity might be due to the unfavorable environment (e.g., lower temperature) of water and its lack of nutrients, which can reduce the ability of some bacteria commonly present in the chicken gut to survive and multiply (McAllister and Topp, 2012). Interestingly, AMU seemed not to have a significant effect on the microbial communities of any of the investigated samples. Indeed, within each same sample type, neither richness nor diversity were affected by AMU. Several studies investigating the effects of AMU on the chicken gut microbiota reported both reduced and increased diversity, as well as no changes in the microbial composition and structure (Schokker et al., 2017; Le Roy et al., 2019; Laconi et al., 2022a). Increased or comparable diversity in the chicken gut after treatments has been previously attributed to the proliferation of resistant bacteria compensating for the loss of non-resistant species (Laconi et al., 2022a). However, in this study, specific taxa neither disappeared nor bloomed in the AMU_group, suggesting a widespread dissemination of resistant bacteria in Cameroonian poultry farms, as supported by the high prevalence of ARGs observed.

### 4.2 High prevalence and different profiles of ARGs among sample types

Aiming to understand the level of resistance and the distribution of ARGs in Cameroonian poultry farms, the prevalence of selected genes conferring resistance to medically important antimicrobials was assessed in drinking water and biofilm collected from drinking water distribution systems and fecal samples by using a culture-independent approach (i.e., real-time PCR). On one hand, the paucity of studies investigating AMR in poultry production in Central Africa (Vougat Ngom et al., 2024a) using culture-independent methods highlights the novelty and potential impact of the present study. On the other hand, it hampers a straightforward comparison between the prevalence of ARGs observed here and previous studies conducted in the same geographical area. However, some considerations can be drawn. In this study, for instance, a higher prevalence of *qnrS* (100%) was detected compared to previous observations, since this gene has been identified in less than 50% of bacteria isolated from chickens, fecal droppings and/or farm environment (Ayandiran et al., 2018; Nchawa et al., 2019b; Kimera et al., 2021; Leinyuy et al., 2023). Moreover, even when adopting a culture-independent strategy (i.e., metagenomics), the level of resistance to (fluoro) quinolones in Ghanaian poultry farms was lower compared to our findings (Jensen et al., 2023). Previous studies failed to identify resistance genes to polymyxins other than *mcr-1* (Nchawa et al., 2019a; Vounba et al., 2019b; Kimera et al., 2021); however, four additional *mcr* genes were detected across the different samples in this study, representing a significant concern for public health. Of further concern is the detection of the *bla_NDM_* gene in feces and biofilm. While genes encoding for carbapenemases (e.g., *bla_OXA_*) have been previously detected in bacteria isolated from poultry farms in African countries (Nchawa et al., 2019b; Vounba et al., 2019a; Aworh et al., 2021), this represents the first ever detection of *bla_NDM_*. Moreover, most samples (64, 95% CI 49.9–79.0%) showed a MDR profile, being resistant to at least three different antimicrobial classes. Since one of main drivers of the emergence of AMR is the use of AMDs (Holmes et al., 2016), the observed high level of resistance could be reasonably attributed to the misuse and overuse of antimicrobials in the farms under investigation. Indeed, while only 7 out of 15 farmers reported using at least one AMD (e.g., norfloxacin, colistin, and/or tylosin), belonging to one of the antimicrobial classes investigated in the 2 weeks prior sampling, antimicrobials were routinely administered for metaphylaxis and prophylaxis in all sampled farms. Supporting the key role played by the selective pressure exerted by antimicrobials on AMR, farms where AMDs were more lately used showed a trend of increased resistance to polymyxins, carbapenems, and multi-drugs. Furthermore, farms scoring high on good practices on AMU were associated (*p* < 0.05) with decreased resistance to polymyxins and MDR. This finding suggests that education of farmers on AMU and the risks related to AMDs overuse and misuse may contribute to the reduction of AMR in poultry production, as previously pointed out by Caekebeke et al. (2021). However, the emergence and persistence of antimicrobial resistant bacteria and genes in the farm environment is a complex phenomenon and factors other than AMU can contribute to it. For instance, since all farms used traditional and/or modern wells as their water source, the high prevalence of ARGs detected in this study may also reflect AMR environmental contamination. Notably, some of these resistance genes (e.g., *ermA*, *ermB*, *oqxA*, and *qnrS*) are capable of persisting and accumulating in fertilized soil (Laconi et al., 2020). ARGs in amended soil can reach waterways and water sources through drainage (Hruby et al., 2016), potentially perpetuating a self-sustained cycle of AMR maintenance in poultry farms and their surrounding environment. For instance, since *mcr-1* was more prevalent (*p* < 0.05) in feces and drinking water compared to biofilm, drinking water may represent a possible source of this ARG. On the other hand, although showing prevalence as high as 40 and 15% in drinking water and biofilm samples, respectively, *mcr-3* was not found in feces. Meanwhile, *bla_NDM_* was detected only in feces and biofilm, with similar prevalence in both sample types, suggesting that biofilm may play a role in its persistence and spread within poultry farms. Overall, these findings seem to further confirm that the dissemination of antimicrobial determinants in the farm environment is a complex phenomenon. While drinking water and biofilm in drinking water distribution systems may represent an important reservoir of resistance genes and bacteria, other sources (e.g., feed, pests, wildlife, and farmers) can contribute to their transmission and proliferation in the chicken gut (Daehre et al., 2017; Apostolakos et al., 2019).

### 4.3 Farmers’ knowledge and behavior toward AMU and perception on AMR

This study revealed that all poultry farmers used AMDs on their farms and the farmers’ knowledge on AMU and AMR was assessed as insufficient. This finding is worrying given that the majority of farmers, besides having an acceptable level of education (most of them were at least at a secondary school level), had more than 6 years of experience in poultry farming. This result can be associated with the low contact of the farmers with animal health specialists due to their limited number and the high fees of their services (Caudell et al., 2019; Tasmim et al., 2023). Indeed, only 40% of farmers obtained veterinary drugs based on a veterinarian prescription, aligning with those of Moffo et al. (2020) and Sawadogo et al. (2023) in the Center Region of Cameroon and Ouagadougou in Burkina Faso, respectively. Furthermore, the lack of awareness of the risk posed by AMR among poultry farmers detected in this study is similar to that detected across the African continent (Al Sattar et al., 2023). Such widespread unawareness may contribute to increasing AMR in African livestock, with a potential impact also on human and environmental health. Surprisingly, the mean score on AMU practices was found appropriate. Moffo et al. (2020) reported similar results in the same study area, whereas an inappropriate AMU was recorded by Chah et al. (2022) in Nigeria. In our study, 80% of farmers used antibiotics for prevention and 50% of the most commonly used antibiotics were medically important. The use of these drugs is of great concern, since they are commonly used to treat severe and life-threatening infections in humans. Misuse of AMDs can heavily contribute to the high level of resistance detected in this study, as also highlighted in a recent review considering different livestock species in Africa (Vougat Ngom et al., 2024a).

Farmers’ risk perception on AMR was inappropriate with only 20% of farmers perceiving that misuse of AMDs can contribute to the emergence of AMR. In addition, less than a quarter was aware that AMR could be harmful for both themselves and their animals. These findings corroborate the results of a previous study on risk perceptions of poultry farmers in Kwara state in Nigeria (Al-Mustapha et al., 2023). Interventions for mitigating AMR in Cameroon should focus on raising awareness on AMR in poultry farmers. This is crucial considering the increasing number of farmers in this sector due the increased consumer demand of chicken meat and eggs. In addition, improving knowledge and awareness of AMU and AMR through targeted and contextualized education, as well as improving legislation and regulations of veterinary drug purchase and use can also contribute to improving the current status.

### 4.4 Biosecurity implementation

The overall level of biosecurity implementation in the studied farms was intermediate. This is probably associated with the lack of strict legislation on farm biosecurity in Cameroon. Farmers’ knowledge and attitude toward biosecurity implementation may also contribute to this result (Klein et al., 2023). Moffo et al. (2022) reported a low biosecurity level in broiler farms in the same study area. The conflict among these results may be attributed to the presence of layer farms in our study. Since layer farms require more investment, farmers might be more aware of the risk and the cost associated with disease occurrence (Islam et al., 2023). Low biosecurity levels in poultry farms have also been reported in other African countries (Waktole et al., 2023). Poor biosecurity in poultry farms has been associated with an increase of animal diseases (Vougat Ngom et al., 2024b) and AMU in Africa (Moffo et al., 2022). A higher level of implementation of internal rather than external biosecurity was recorded in this study. This suggests that farmers put more effort to control than to prevent the occurrence of infectious diseases in their farms. This finding suggests a need for effective training, advising and communication on biosecurity for farmers. Similar findings have also been found in poultry farms in Africa (Moffo et al., 2022; Waktole et al., 2024), Europe (Laconi et al., 2023; Souillard et al., 2024), and Asia (Tanquilut et al., 2020; Mirzaie et al., 2023). The higher implementation of internal biosecurity may be associated with the farmers’ knowledge and perception on biosecurity and a stronger link between internal biosecurity and disease occurrence. This result highlights the need for improving biosecurity legislation and farmer’s awareness on biosecurity in Cameroon.

### 4.5 Limitations of the study

In the present study, the antimicrobial resistance, the level of biosecurity implementation, the farmers’ knowledge, attitude, and practices toward AMU, and their knowledge and perception of AMR risk were investigated in poultry farms in the Center region of Cameroon. While the results of this study cannot be generalized to the entire country, due to the limited sample size, they provide valuable insights for future research. Furthermore, while this study represents the first attempt to investigate AMR and microbial community composition in Cameroonian farms using a culture-independent approach and advanced molecular biology techniques (i.e., real-time PCR and next-generation sequencing), limitations in DNA quantity and quality may have hindered the quantification of ARGs and the characterization of microbiota at taxonomic levels higher than family.

## 5 Conclusion

Considering the complexity of AMR in livestock production and its impact on environmental and human health, adopting culture-independent and one health approaches are required to understand the prevalence and diversity of ARGs in poultry farms. However, due to financial constraints, inadequate laboratory facilities, and/or lack of trained personnel, implementing these methods may be challenging in developing countries. Efforts should be made to support cooperation and knowledge transfer between high-and low-income countries. In the present study, more than half of the samples showed MDR and carried ARGs for last resort antimicrobials (i.e., *mcr* and *bla_NDM_* genes), posing thus a great concern, not only for animals, but also for public and environmental health. Indeed, most farmers declared that only polymyxins seem to be still effective for treating bacterial infections in their farms. The use of AMDs, including highest priority critically important antimicrobials, for metaphylactic and prophylactic treatments, may have contributed to the high prevalence of ARGs in Cameroonian poultry farms. Notably, good AMU practices appear to be associated with a reduction in polymyxins resistance and MDR. Farmers’ knowledge and attitude toward AMU, and their knowledge and perception of the risks posed by AMR, have been found to be scarce. This, coupled with an overall modest level of biosecurity compliance, needs improvements to reduce the AMDs use and mitigate the AMR risk effectively. Education programs for farmers, along with the development of more stringent and enforced regulations on AMU and biosecurity at national level, are strongly advocated. Overall, the study underscores the need for improved practices and regulations to tackle AMR in the poultry sector in Cameroon. By addressing these issues through education, better biosecurity practices, and international cooperation, significant progress can be made in controlling AMR and ensuring sustainable poultry production.

## Data Availability

The datasets presented in this study can be found in online repositories. The names of the repository/repositories and accession number(s) can be found at: https://www.ncbi.nlm.nih.gov/, PRJNA1123609.

## Footnotes

^1^ https://www.microbiomeanalyst.ca

^2^ www.graphpad.com

## Supplementary material

SUPPLEMENTARY MATERIAL 1 Copy of the questionnaire used to collect information regarding farms’ characteristics, biosecurity practices, farmers’ knowledge, attitudes and practices on antimicrobial use (AMU), and knowledge and risk perception on antimicrobial resistance (AMR).

SUPPLEMENTARY MATERIAL 2 DNA concentration (ng/μl) of the analyzed samples.

SUPPLEMENTARY MATERIAL 3 The list of assays, primers pairs, melting temperatures, and positive controls used in the study.

SUPPLEMENTARY MATERIAL 4 α-and β-Diversity according to sample type and antimicrobial use.

SUPPLEMENTARY MATERIAL 5 Linear discriminant analysis (LDA) scores of LDA effect size (LEfSe) comparison analysis between fecal, drinking water, and biofilm samples. Red shading depicts bacterial taxa significantly higher in a given sample type.

SUPPLEMENTARY MATERIAL 6 Prevalence (%) of ARGs according to sample type and antimicrobial use. Whiskers represent 95% CI.
