# Supplementary material for: Resistance to medically important antimicrobials in broiler and layer farms in Cameroon and its relation with biosecurity and antimicrobial use
Source: Front Microbiol. 2025 Jan 15;15:1517159. doi: 10.3389/fmicb.2024.1517159 (PMC11774882; doi:10.3389/fmicb.2024.1517159)
Supplement: SUPPLEMENTARY MATERIAL 1 — Copy of the questionnaire used to collect information regarding farms’ characteristics, biosecurity practices, farmers’ knowledge, attitudes and practices on antimicrobial use (AMU), and knowledge and risk perception on antimicrobial resistance (AMR). [file Data_Sheet_1.PDF]

Machine Translated by Google

QUESTIONNAIRE

I-Information on the operation

1. Card number

2. Date of visit

3. Region

- Centre
- West
- Littoral
- North
- Adamawa

4. Loss

5. District

6. Locality

7. GPS coordinates

II- Information on the operator

8. Telephone number

9. Gender

- Male
- Female

10. Level of education

- None
- Primary
- Secondary
- Superior

11. Age

- 20-29
- 30-39
- 40-49
- 50-59
- 60-69

12. Poultry training

Yes No

13. Livestock breeding as main activity

Yes No

14. If not, what is your main activity?

15. Years in the field as a breeder

0-5 years 6-11 years 12-17 years

18-23 years old

Over 23 years old

16. Do you have a health worker responsible for monitoring the farm?

Yes No

17. What is his qualification?

- Veterinarian
- Veterinary nurse
- Livestock technician
- Agricultural engineer
- Others________________

18. How often does he visit your farm?

?

- 1 visit/month
- 3 visits/month
- in case of problem
- others ________

19. How long has he known your business?

- 0-5 years
- 6-11ans
- 12 years and over

III-Information on the operation

20. Speculation

Chair Bridge

Mixed

21. Livestock size

- Less than 1000
- 1001-5000
- 5001-10000
- 10001 and more

22. Age of subjects (weeks)

0-4 5-9 10-14

15-19

20+

Machine Translated by Google

23. Recurring diseases observed on the farm

- ☐ Respiratory ☐ Digestive ☐ cannibalism
- ☐ Urogenital ☐ Locomotor ☐ Cutaneous
- ☐ Nervous

You can check multiple boxes.

24. How do you diagnose diseases?

- ○ Following symptoms Laboratory ○ test
- ○ don't know

25. Density

- ○ Less than 6 ○ 6-9 + 9 ○

26. What are the symptoms that indicate a disease? disgestive?

- ☐ Abnormal droppings You ☐ Decrease in consumption

can check multiple boxes.

27. What are the symptoms that guide you towards a respiratory disease?

- ☐ Snoring
- ☐ Decrease in consumption
- ☐ Cough
- ☐ Shell modification
- ☐ Weakness
- ☐ Bridge kick
- ☐ Eternuements
- ☐ Runny nose
- ☐ Swollen head

You can check multiple boxes.

28. What are the symptoms that guide you towards a nervous disease?

- ☐ Abatement ☐ Somnolence ☐ Torticollis

You can check multiple boxes (maximum 2).

29. What are the symptoms that guide you towards a locomotor problem?

- ☐ Leg Deformation You can ☐ Boxes

check multiple boxes.

30. How common are digestive diseases in your farm?

- ○ Very high ○ Raised ○ Average
- ○ weak ○ very weak

31. How common are respiratory diseases in your farm?

- ○ Very high ○ Raised ○ Average
- ○ weak ○ very weak

32. How common are nervous diseases?

- ○ Very high ○ Raised ○ Average
- ○ weak ○ very weak

33. How common are locomotive problems in your farm?

- ○ Very high ○ Raised ○ Average
- ○ weak ○ very weak

IV- Knowledge on the use of antibiotics and antibiotic resistance

34. Have you ever heard of antibiotics?

- ○ Yes No○

35. What do you think it is?

- ○ ________ ________

36. What are the different types of antibiotics that you know?

- ☐ Bacteriostatic ☐ Bactericide ☐ don't know

You can check multiple boxes (maximum 2).

37. Have you ever heard of antibiotics? bacteriostatic or bactericidal?

- ○ Yes No○

38. What is a bacteriostatic antibiotic according to you?

- ○ Inhibits bacterial growth ○ Kills bacteria
- ○ Don't know

39. What is a bactericidal antibiotic according to you?

- ○ Inhibits the growth of bacteria ○ Kills bacteria
- ○ Don't know

40. Which of the following antibiotics are bacteriostatic according to you?

- ○ Penicilins, Aminosides, Colistin, Enrofloxacin, Fluméquine, Tylosine
- ○ Oxytétracycline, Doxycycline, Sulfamids-Trimethoprim
- ○ Don't know

41. Which of the following antibiotics are bactericides according to you?

- ○ Penicilins, Aminosides, Colistin, Enrofloxacin, Fluméquine, Tylosine
- ○ Oxytétracycline, Doxycycline, Sulfamids-Trimethoprim
- ○ Don't know

42. What germs are affected by antibiotics? are effective?

- ○ Bacteria ○ Other mushrooms ○ Virus
- ○ Parasites ○ ○ Don't know

43. What type of germs are antibiotics not effective against? effective?

- ○ Bacteria ○ Mushrooms ○ Virus
- ○ Parasites ○ Others ○ Don't know

Machine Translated by Google

44. What do you use antibiotics for?

- ☐ Preventive
- ☐ Metaphylactic
- ☐ Curative
- ☐ Boosting growth
- ☐ prevention of secondary infections after the onset of a viral disease
- ☐ After vaccination You can

check several boxes.

45. What antibiotics do you use to treat respiratory diseases?

☐ Oxytetracycline Enrofloxacine ☐ Streptomycine ☐ Flumequine
☐ Norfloxacine ☐ Amoxicillin ☐ Doxycycline
☐ ☐ Lincomycine ☐ Spiramycine
☐ Tylosin ☐ Colistine

You can check multiple boxes (maximum 9).

46. What antibiotics do you use to treat digestive diseases?

☐ Norfloxacine ☐ Sulfonamides
☐ Flumequine ☐ Streptomycin
☐ Oxytetracycline ☐ Enrofloxacine
☐ Colistine ☐ Doxycycline
☐ Amoxicillin

You can check multiple boxes (maximum 8).

47. What antibiotics do you use for treat viral diseases?

☐ Oxytetracycline ☐ Norfloxacine ☐ Flumequine
☐ Colistine ☐ Doxycycline ☐ Enrofloxacine
☐ None

You can check multiple boxes.

48. What are the problems associated with misuse? antibiotics in livestock farming?

- ☐ Residues in foodstuffs
- ☐ Antimicrobial resistance
- ☐ drugs in the environment
- ☐ Don't know

You can check multiple boxes (maximum 3).

49. What are the factors that affect the quality of an antibiotic?

☐ High temperature ☐ heat ☐ poor storage
☐ sun

You can check multiple boxes.

50. Are antibiotics also used as painkillers?

○ Yes No ○

51. Can interactions between multiple antibiotics used at the same time reduce the effectiveness of some antibiotics in treating bacteria?

○ Yes No ○ Don't know ○

52. Have you ever heard of resistance to antibiotics?

○ Yes No ○

53. If yes, what do you think it is?

○ ______________
____

54. In your opinion, what are the practices that lead to the emergence of resistance to antibiotics?

- ☐ Use of antibiotics as a preventative measure
- ☐ overdose of antibiotics use of
- ☐ expired drugs use of street drugs
- ☐
- ☐ underdosing of antibiotics self-
- ☐ medication
- ☐ Don't know

You can check multiple boxes.

55. Can the use of antibiotics as anti-stress or preventive measures induce resistance?

○ Yes No ○ Don't know ○

56. Can using the same antibiotic over a long period of time induce resistance?

○ Yes No ○ Don't know ○

57. Can the rational use of an antibiotic (correct dosage) lead to the development of resistance?

○ Yes No ○ Don't know ○

58. Have you ever heard of an antibiogram?

○ Yes No ○

59. If yes, what do you think it is?

○ __________

60. In what circumstances did you hear about the antibiogram?

- ○ Training school
- ○ seminar
- ○ Chatting with other farmers
- ○ Discussion with the veterinarian
- ○ Hospital (Human Medicine)

V- Attitudes

61. How do you feel about your level of knowledge about antibiotics?

○ 1 ○ 2 ○ 3 ○ 4 ○ 5

1) very confident, 2) confident, 3) neutral (no idea), 4) not very confident; not at all confident

62. How often do you seek the advice of a veterinarian? before administering antibiotics? sometimes

○ Never ○ most times
○ half the time ○
○ Always

Machine Translated by Google

63. Are you confident that the use of antibiotics on a long period may induce resistance to antibiotics?

○ 1 ○ 2 ○ 3 ○ 4 ○ 5

1) Strongly agree; 2) Agree; 3) Neutral; 4) Disagree; 5) Do not don't know ;

64. Do you agree that the use of antibiotics as growth promoter or as a preventative measure can induce antibiotic resistance?

○ 1 ○ 2 ○ 3 ○ 4 ○ 5

65. Do you agree that the rational use of antibiotics can help reduce resistance to antibiotics?

○ 1 ○ 2 ○ 3 ○ 4 ○ 5

66. Are you confident that the misuse of antibiotics can induce resistance which contaminate the environment and can be transmitted to the animal and the man?

○ 1 ○ 2 ○ 3 ○ 4 ○ 5

67. In recent years, have you followed a training on the proper use of antibiotics and concept of antibiotic resistance

○ Yes ○ No

VI- Practices

68. Who judges the need for antibiotic treatment in your farm?

○ Veterinarian
○ Veterinary nurse
○ Owner
○ farmer
○ others ___________

69. Where do you get your medication?

☐ Veterinary pharmacy ☐ Local market
☐ Health worker ☐ Fellow breeder
☐ Provençal

You can check multiple boxes.

70. How do you choose your antibiotics?

☐ Personal choice
☐ Cost
☐ availability
☐ Efficiency
☐ Prescription by the veterinarian
☐ prescription by the local market seller
☐ Prescription by the feed supplier

You can check multiple boxes.

71. Are your subjects on antibiotics or anti-stress medication? this moment?

○ Yes ○ No ○ Don't know

72. If yes, which ones?

○ Amine total ○ Doxycycline ○ Amoxixilline
○ Enrofloxacine ○ Oxytetracycline ○ Lincomycine
○ Colistine

73. Who regularly administers your care?

☐ Owner
☐ Veterinarian
☐ Employees
☐ Others_______________________________

You can check multiple boxes.

74. How do you determine the amount/dose? antibiotic for your subjects

☐ According to medical instructions
☐ Weighing the subjects
☐ By estimating the weight of the subjects
☐ According to the age of the subjects
☐ Don't know
☐ Others

____

You can check multiple boxes.

75. When do you stop treatment?

○ Disappearance of symptoms
○ Respect of deadlines recommended

76. If treatment fails, repeat the same molecule?

○ Always until complete healing
○ Often
○ Never (I always try an alternative)
○ Don't know

77. How often at most do you apply the same medication before changing to a possible alternative

○ 1J ○ 2J ○ 3J ○ 4J ○ 5J ○ 6J ○ 7J

78. Should the dose be increased to increase effectiveness? ?

○ Yes ○ No

79. If symptoms persist after a first treatment, what is your attitude?

○ Increase the dose of the same treatment
○ Extend the duration of the same treatment
○ Change molecule
○ Prescribe a combination of antibiotics
○ Use of the laboratory
○ diagnosis (antibiogram)
○ Commercialisation
○ Native treatment

Machine Translated by Google

80. Do you respect the processing times prescribed by the veterinarian

○ Always ○ Never ○ Sometimes

81. If not, why?

○ Economic loss ○ Don't know
○ ______

82. Do you add antibiotics or anticoccidials in the food?

○ Yes No○

83. If yes, which ones?

☐ Sulfonamides ☐ Penicillins ☐ Tetracyclines

You can check multiple boxes.

84. If yes, what quantity for 100Kg or 1000 kg?

○ ______________

85. Do you use antibiotics in the initial phase of treatment? chicks?

○ Yes No○

86. If yes, which ones?

☐ Colistine ☐ Amoxicillin ☐ Oxytetracycline
☐ Enrofloxacine ☐ Norfloxacine ☐ Doxycycline

You can check multiple boxes (maximum 4).

87. What dosage do you apply in case of treatment? preventive?

○ Recommended dosage ○ Dose reduction

88. What is the duration of preventive treatment (days)?

○ 1 J ○ 2J ○ 3J ○ 4 J ○ 5J

89. What is the interval between preventive treatments?

○ After every week 3 weeks ○ 2 weeks
○ ○ 4 weeks

90. What antibiotics do you use as a preventive?

☐ Colistine ☐ Oxytetracycline ☐ Enrofloxacine
☐ Tylosine ☐ Flumequine ☐ Doxycycline
☐ Lincomycine ☐ Sulfonamides ☐ Norfloxacine
☐ Amoxicilline

You can check multiple boxes (maximum 4).

91. Do you often combine several antibiotics to increase efficiency?

○ Yes No○

92. If yes, which ones?

☐ Colistine + Oxytétracycline
☐ Tylosine + Doxycycline
☐ Enrofloxacine + Colistine
☐ Amoxicilline + Tylosine
☐ Colistine + Norfloxacine
☐ Colistine + Doxycycline

You can check multiple boxes (maximum 4).

93. If yes, how often?

○ Always sometimes ○ ○ very rarely

94. Do you combine antibiotics with diuretics? to administer to chickens?

○ Yes No○

95. If yes, which ones?

☐ Oxytetracycline ☐ Amoxicillin ☐ Enrofloxacine

You can check multiple boxes (maximum 2).

96. How often do you do this?

○ Always
○ Sometimes
○ very rarely
○ Others ____________

97. Do you often combine oxytetracycline or doxycycline with minerals to administer to chickens?

○ Yes No○

98. If yes, how often?

○ Always sometimes ○ ○ very rarely

99. What is the antibiotic you use the most?

○ Oxytétracycline Colistine ○ Doxyxycline ○ Norfloxacine
○ Lincomycine ○ Amoxicillin ○ Enrofloxacine
○

100. Which antibiotics do you prefer the most?

?

○ Oxytetracycline ○ Colistine ○ Tylosine
○ Doxycycline ○ Enrofloxacine ○ Spiramycine
○ Norfloxacine ○ Amoxicillin ○ Sulfonamides

101. Among the antibiotics you use, which ones do not seem more efficient to you on your farm?

☐ Oxytetracycline Doxycycline ☐ Enrofloxacine ☐ Norfloxacine
☐ Amoxicilline ☐ ☐ Tylosine

You can check multiple boxes (maximum 2).

102. What do you do with dead chickens after treatment? antibiotics?

○ Cremated
○ thrown into a pit
○ thrown into a trash can
○ consumed by employees
○ plucked and sent to the boss (marketed)
○ throw to the dog

103. During treatment do you continue the collection and sale of eggs or sale of chickens??

○ Yes No○

104. If not, what do you do with the eggs collected during the treatment???

○ ___________

Machine Translated by Google

105. When treatment is not effective, what should you do? you have any topics??

- sale
- slaughter
- Others___________________

106. How do you manage droppings?

- Biogas
- Sale
- spreading in your fields
- manufacturing of the Compos
- others___________________________________

107. Between biosecurity measures and medicines, which ones do you think are more effective for the protection of animals against pathologies?

- Biosafety Medicines

108. What do you do with expired medications?

- Return to pharmacy Pour into nature
- Keep away from heat
- Administer to animals
- No expiration date

VII- Risk perception by breeders

109. Can misuse of antibiotics cause cause problems for man?

- Yes No Don't know

110. Do you think that antibiotic resistance in Breeding can be a health problem human?

- Yes No Don't know

111. Do you think that antibiotic resistance can constitute a problem for animal health?

- Yes No Don't know

112. Do you think that antibiotic resistance can constitute a problem for the environment?

- Yes No Don't know

113. What is your risk level of being infected? by antibiotic-resistant germs from the farm?

- 1 - 2 - 3 - 4 - 5

1) very low, 2) low; 3) medium; 4) high; 5) very high

114. What is the risk level for your subjects to be infected with antibiotic-resistant germs?

- 1 4 - 2 - 3 - 5

115. What is the risk level for germs? antibiotics to spread in the environment?

- 1 - 2 - 3 - 4 - 5

116. Do you think that germs resistant to Antibiotics used in livestock farming can be transmitted to the man?

- Yes No Don't know

117. If yes, by what means?

- Consumption
- Contact direct
- Dust inhalation Don't know

You can check multiple boxes (maximum 3).

118. Do you know what residues are? of antibiotics in DAOA?

- Yes No

119. If yes, what do you think it is?

- ________________
____

120. Do you think that the drug residues that you use can end up in food?

- Yes No Don't know

121. If yes, do you think these residues are dangerous? for man??

- Yes
- Non
- I don't know
- other _____________

122. Do you know what is meant by maximum limit? residues in a DAOA?

- Yes No

123. If yes, what do you think it is?

- ________________
____

124. Do you know what the waiting period is? antibiotic therapy?

- Yes No

125. Do you respect the waiting period prescribed by the manufacturer?

- Always
- sometimes
- Never

126. Do you often take antibiotics during sales?

- Yes No

127. Do you often use the following products during the laying period?

- Doxycycline
- Ampicillin
- Sulfonamides
- Enrofloxacine
- Flumequine
- Tylosine
- Lyncomycine
- Oxytetracycline

128. Did you know that these products should not be used in chickens during the laying period and whose eggs are intended for human consumption?

- Yes No

129. Would you be able to reduce the use of antibiotics in your breeding?

- Yes No

130. Under what condition?

- ☐ Less diseases
- ☐ Alternatives to treatments
- ☐ State subsidies and regulations
- ☐ Qualities of available strains
- ☐ Less staff
- ☐ Effective biosecurity

You can check multiple boxes (maximum 2).

131. Would you be willing to receive training? in-depth study on the proper use of antibiotics and antibiotic resistance?

○ Yes N○

VIII- External Biosecurity (100)

VIII-1. Location of the farm (14)

132. Is there a watercourse or a pool of stagnant water within a radius of 200 meters?

○ Yes (0) N○ (5)

133. Is there a source of nuisance within a 50m radius? sound

○ Yes (0) N○ (3)

134. If yes, please specify?

- ☐ Aerodrome
- ☐ Busy road
- ☐ Rail
- ☐ Others _____

NB: question 134 is not weighted

135. How far is the nearest poultry farm? <100m (0) >1km (6)

○ ○ 100-500m (3) ○ 500-1km (4) ○

VIII-2. Acquisition of subjects (28)

136. Do your day-old chicks come from one hatchery or several?

- ○ Always from a single incubator (10)
- ○ Sometimes from several incubators (7)
- ○ Still from several hatcheries (0)

137. Are means of transport always disinfected? before any delivery?

○ Always (10) ○ Quelqefois (9) ○ never (0)

138. Do chick drivers and loaders have a specific equipment provided by the farm when loading the eggs?

○ Yes (8) ○ Sometimes (7) N○ (0)

VIII-3. Water, food and equipment supply (19)

139. What type of water is used for watering the subjects?

☐ CDE (8)	☐ Drilling (0)
☐ Wells (0)	☐ capture (0)
☐ Draw from a river (0)	☐ others (0)

NB: farms that responded by CDE to question 141 are not concerned by questions 142, 143

140. Is this water treated?

○ Yes N○

Farms that answered “no” to this question are not affected by question 141

141. If yes, how often?

○ Every day (3) 1	○ 1 time/week (2) 1
○ time/month (1)	○ time every 6 months (0)
○ 1 time/year (0)	○ Others (0)

142. How often is drinking water analyzed on your farm?

○ Every year (2)	○ Every 2 years (1)
○ Every 2 years (05)	○ Never (0)
○ Others (0)	

143. Where does your food come from?

○ Approved Manufacturer Other ○ As uto-fabrication ○

Question 143 is not weighted

144. Are the food bags always properly sealed?

○ Yes N○

Machine Translated by Google

145. Where are the food bags stored?

- Outdoors (0)
- In livestock buildings (0)
- In a store (1)
- In the sanitary airlock (0)
- In the family home (0.5)
- Others (0)

146. Are the feed bags in contact with the ground?

Yes No

147. Does the use of food comply with the principle FIFO (first in, first out)?

Yes No

148. What do we do with the alveoli?

Reused (0) Thrown (2) Others (0)

149. What do you do with egg cartons?

Reused (0) Thrown (2) Others (0)

VIII-4. Entrance of visitors and staff (21)

150. Are there any biosecurity measures posters? intended for visitors to the poultry farm?

Yes (1) No (0)

151. Is there a maximum visiting time provided for the visitors?

Yes (1) No (0)

152. Do the staff have specific work attire?

Yes No

153. if yes which one?

- Outfit + boots (4)
- Boots only (2) Outfit only (2)

NB: question 153 is only relevant if the answer is "yes". » to question 152

154. Are visitors and staff required to wash and disinfect their hands upon entering exploitation?

Yes (4) No (0)

155. Are there any employees who have their own breeding?

Yes (0) No (3)

156. Are there employees who work in other farms?

Yes (0) No (3)

VIII-5. Infrastructures and biological vectors (18)

157. How many buildings are there on the farm?

1 (2) 2 (0) 3 (0) 4 (0) 5 (0)
6-10 (0) >10 (0)

NB: questions 158, 159 and 160 are only relevant in the event of response other than modality “1” of question 159

158. What is the distance between groups of buildings?

☐ <10m (0) ☐ 10-20m (0,5) >25m (1)

You can check multiple boxes.

159. What is the distance between buildings?

<1m (0) 2-10m (0,5) 10-15m (0,75)
15-20m (0,8) >20m (1)

160. What is the orientation of the livestock buildings?

- In parallel (1)
- And diagonal (0)
- Square (0)
- Messy (0)
- Others (0)

161. Is the farm fully fenced?

Yes (2) Partially (1) No (0)

162. The upper side parts of the buildings Are breeding operations covered?

Yes (0.5) Partially (0.25) No (0)

163. What materials are they made of?

☐ Bamboos (0.25) ☐ Fences (0.25)
☐ Concrete blocks (0.25)

This question is only relevant if the answer is "yes" or " partially” to question 164

164. What materials are the side parts made of? inferior?

Boards (0.5) Concrete blocks (0.75) Bamboos (0.5)
Others (0)

165. If in concrete blocks, the lower side parts of the Are the walls of the livestock buildings concreted?

Yes (0.25) Non (0)

166. Are the floors of the livestock buildings concreted?

Yes (1) No (0)

167. Are the surroundings of the farm paved?

Completely (1) Partially (0.5) Non (0)

168. Drainage routes for waste water, rainwater and Are there any others built around the chicken coops?

Yes (1) No (0)

169. Is there a skylight?

Yes No

Machine Translated by Google

170. If yes, what is it like?

○ Simple (1) ○ Double (2)

171. Are there any bird protection devices at the skylight?

○ Yes (1) No○ (0)

172. Are your chicken coops locked?

○ Yes (1) No○ (0)

173. Are there signs prohibiting access to foreign persons?

○ Yes (1) No○ (0)

174. Do your pets have access to the outdoors?

○ Yes (0) No○ (1)

175. Are there vermin and harmful animals in the exploitation?

○ Enough (0) ○ Few (0) ○ Not at all (1)

176. Is there a program to control these animals?

○ Yes (1) No○ (0)

NB: this question is only relevant if the answer to question 176 is "quite a bit" or "little bit".

177. Are other chickens raised outside?

○ Yes (0) No○ (1)

178. If yes, are these chickens vaccinated?

○ Yes (1) No○ (0)

179. Are other species raised on the farm?

○ Yes (0) No○ (1)

IX. Internal biosecurity (100)

IX.1. Disease management (15)

180. Is there a vaccination protocol?

○ Yes (2) No○ (0)

181. Is the vaccination and monitoring protocol being followed?

○ Fully (2) ○ Partially (1) ○ Never (0)

182. Is the status of the farm regularly checked?

○ Yes (2) No○ (0)

183. What is the density of animals in the buildings?

○ <6-8 animals/m² (2) ○6-8 animals/m² (2)

○ >6-8 animals/m² (0)

184. Are there animals of different ages in the breeding rooms?

○ Yes (0) No○ (2)

185. Are there animals of different ages present in the farm?

○ Yes (0) No○ (2)

186. Is there a veterinary dispensary on the farm?

○ Yes (0) No○ (3)

187. If yes, how far is it from livestock buildings? <1m (0)

○ 10-40m ○ 1-5m (0.5) ○ 6-10m (1)

○ (2) ○ 40-100m (2) ○ >100m (2)

IX. 2. Cleaning and disinfection (25)

188. Is it always the same disinfectant that you use during disinfection operations?

○ Yes (1) No○ (0)

189. Are there disinfection devices (rotoluve) for vehicles at the entrance to the farm?

○ Yes (1) No○ (0)

190. Are the pipes cleaned often?

○ Yes (1) No○ (0)

191. If yes, how often?

○ Every week (1) 1 time ○ Every month (0.75)

○ per year (0.5) at ○ 1 time every 02 years (0)

○ the end of the strip (0)

192. Is there a water filter?

○ Yes (1) No○ (0)

193. How often are the water troughs cleaned?

○ Every day (2) 1

○ time/2 days (1.5)

○ every week (1) 1

○ time/2 weeks (0.5) 1

○ time/month (0) ? 6.Never (0) Others (0)

194. Are the buildings washed after each shift?

○ Yes (2) No○ (0)

195. Are buildings disinfected after each band?

○ Yes (2) No○ (0)

Machine Translated by Google

196. Are the feeders washed and disinfected regularly?

- Daily (1)
- Weekly (0.75)
- Monthly (0.5)
- At the end of the strip (0)
- Never (0)

197. Are occupied and unoccupied areas washed and disinfected after each shift?

Yes (1) No (0)

198. How long is the crawl space in days? 14 days (0.5)

- <14 days (0)
- 14-30 days (1)
- >1 month (2)

199. Is the farm status (hygienogram) carried out after the crawl space?

Yes (1) Sometimes (0.5) No (0)

200. Is there a sanitary airlock?

Yes (1) No (0)

201. Is this SAS separated into healthy and dirty compartment?

Yes (1) No (0)

202. Are there changing rooms with toilets in the sanitary locker? specific clothes and shoes?

Yes (1) No (0)

203. Is there a functional footbath at the entrance to the farm?

Yes (2) No (1)

204. Is there a functional footbath at the entrance to the livestock buildings?

Yes (2) No (0)

205. Is the footbath solution protected from the elements?

Yes (1) No (0) This question is only relevant if the answer to questions 203 or 204 is "yes".

206. How often is the footbath solution changed?

- Every day (1)
- Every 2 days (0.75)
- Every week (0.5)
- When contamination is visible (0)
- Others (0)

This question is only relevant if the answer to questions 206 or 207 is "yes".

IX-3. Materials and precautions between compartments (32)

207. Is there more than one building on the farm?

Yes (0) No (24)

208. Is there specific equipment for each building on the farm if the farm has more than one building?

Yes (12) No (0)

This question is only relevant if the answer to question 207 is "yes".

209. Is there a protocol for cleaning and disinfecting equipment after use and is it appropriate?

Yes (8) No (0)

210. Is there specific staff for each building?

Yes (12) Non (0)

This question is only relevant if the answer to question 207 is "yes".

IX-4. Removal of animals, litter and carcasses (28)

211. What do you do with the deaths in the chicken coops?

- Cremated (11)
- Thrown into a pit (11)
- Thrown in the trash (7)
- Consumed on site (0)
- Stored in a safe place (5)
- Plucked and sent to the boss (0)
- Marketed)(0)
- Dog sheds (0)

NB: questions 214, 215 and 216 are only relevant if the answer to question 202 is "stored in a safe place".

212. Is this place safe from other animals?

Partially (1) Completely (2) No (0)

213. Is this place regularly washed and disinfected?

- After each collection (2)
- Sometimes (1)
- Never (0)

214. Is this place refrigerated?

Yes (2) No (0)

215. Are gloves worn when handling carcasses and hands disinfected afterwards?

Always (7) Sometimes (4) Never (0)

216. Is there a place for storing litter on the farm?

Yes (0) No (10)

Machine Translated by Google

217. If yes, where is it located?

- ○ In a room inside the farm (5)
- ○ Extracted from the exploitation (6)
- ○ A l'air
- ○ Free in exploitation (0)
- ○ Others (0)

Total biosecurity

218. Internal biosafety note

○ ________________

219. External biosafety note

○ ________________

220. Total biosafety note

○ ________________

221. Farm class

○ C1 ○ C2 ○ C3 ○ C4
