# Supplementary material for: Resistance to medically important antimicrobials in broiler and layer farms in Cameroon and its relation with biosecurity and antimicrobial use
Source: Front Microbiol. 2025 Jan 15;15:1517159. doi: 10.3389/fmicb.2024.1517159 (PMC11774882; doi:10.3389/fmicb.2024.1517159)
Supplement: SUPPLEMENTARY MATERIAL 2 — DNA concentration (ng/μl) of the analyzed samples. [file Presentation_1.PPTX]

## Slide 1
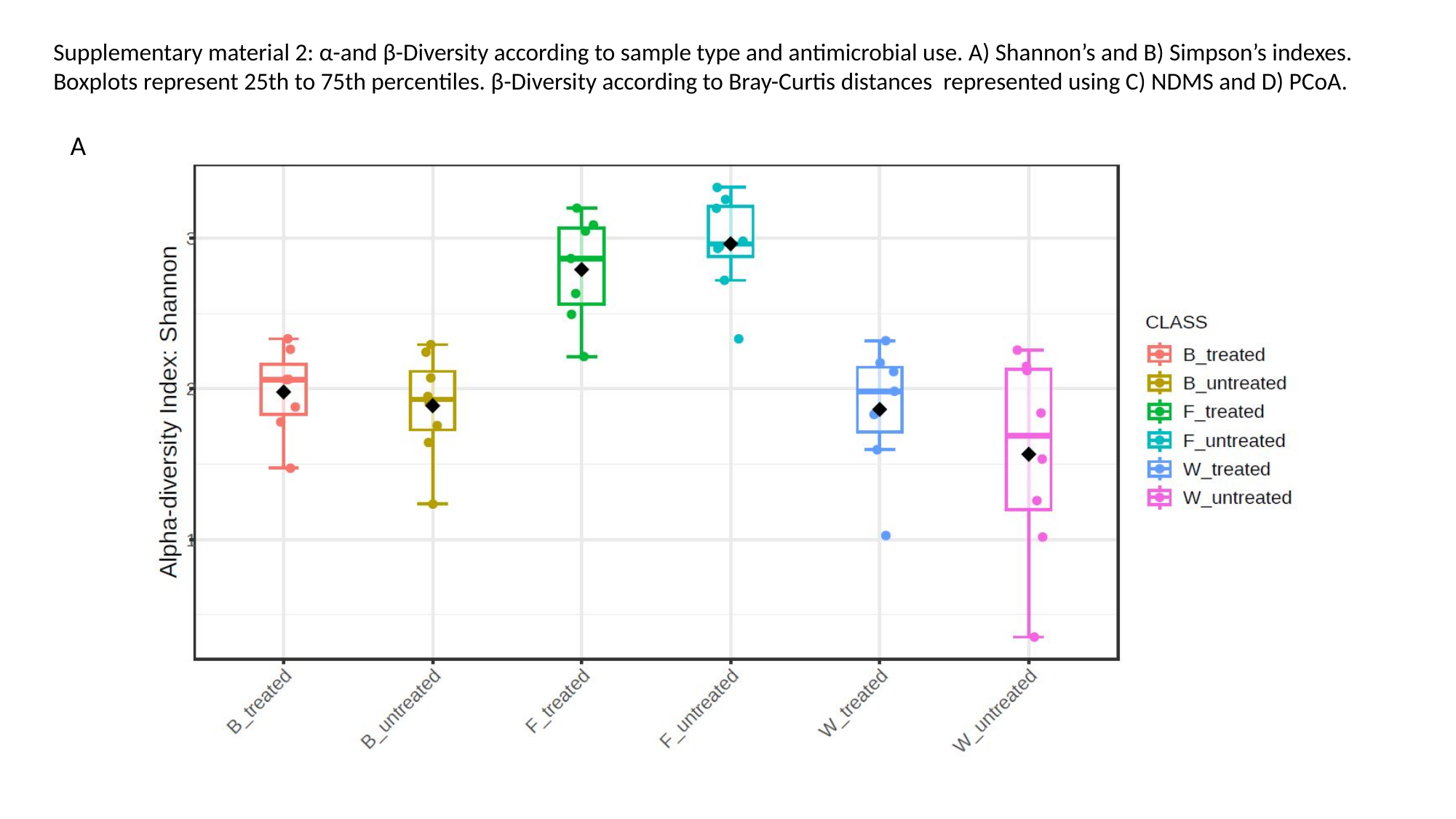

Supplementary material 2: α-and β-Diversity according to sample type and antimicrobial use. A) Shannon’s and B) Simpson’s indexes. Boxplots represent 25th to 75th percentiles. β-Diversity according to Bray-Curtis distances represented using C) NDMS and D) PCoA.
A

## Slide 2
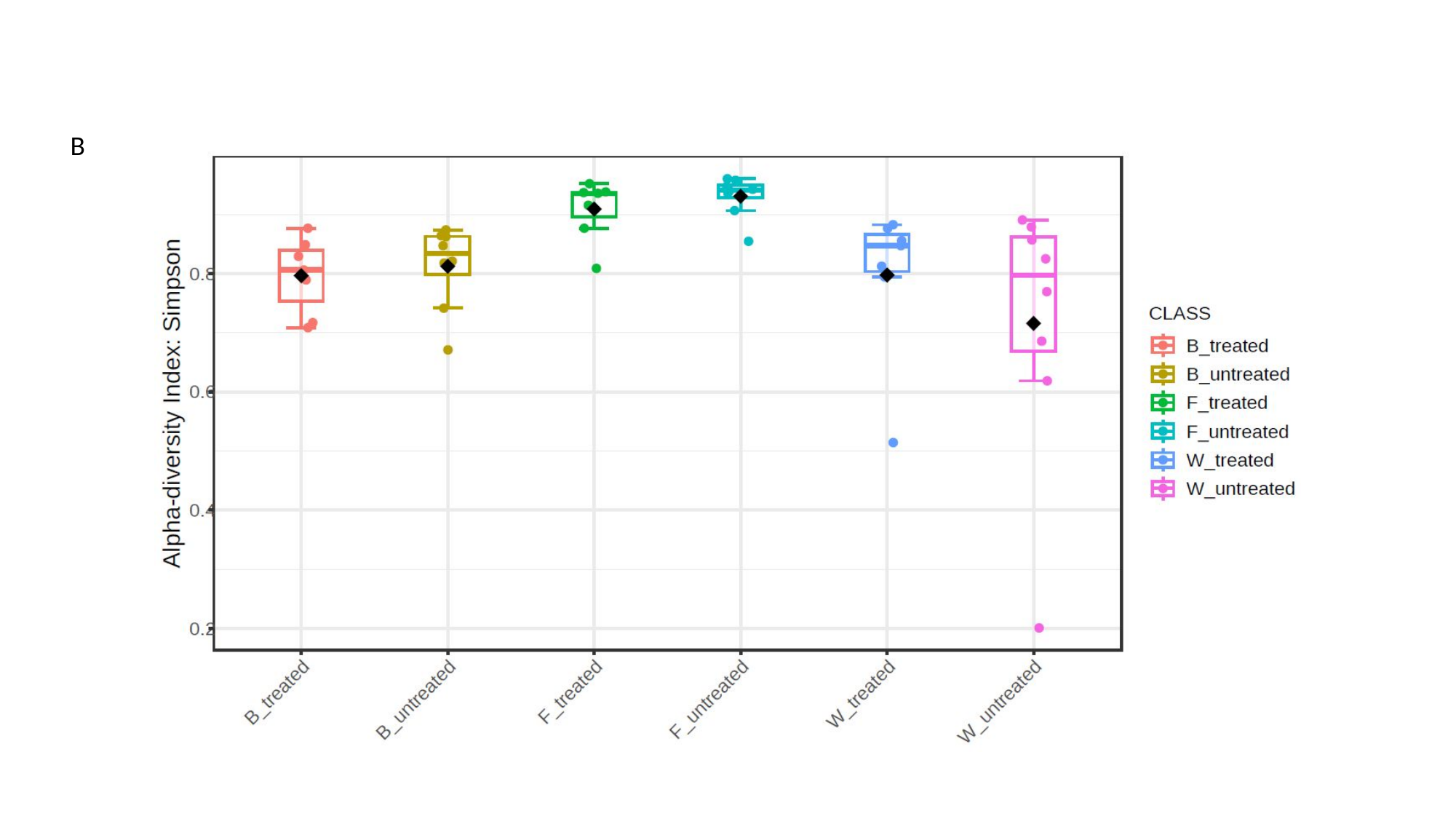

B

## Slide 3
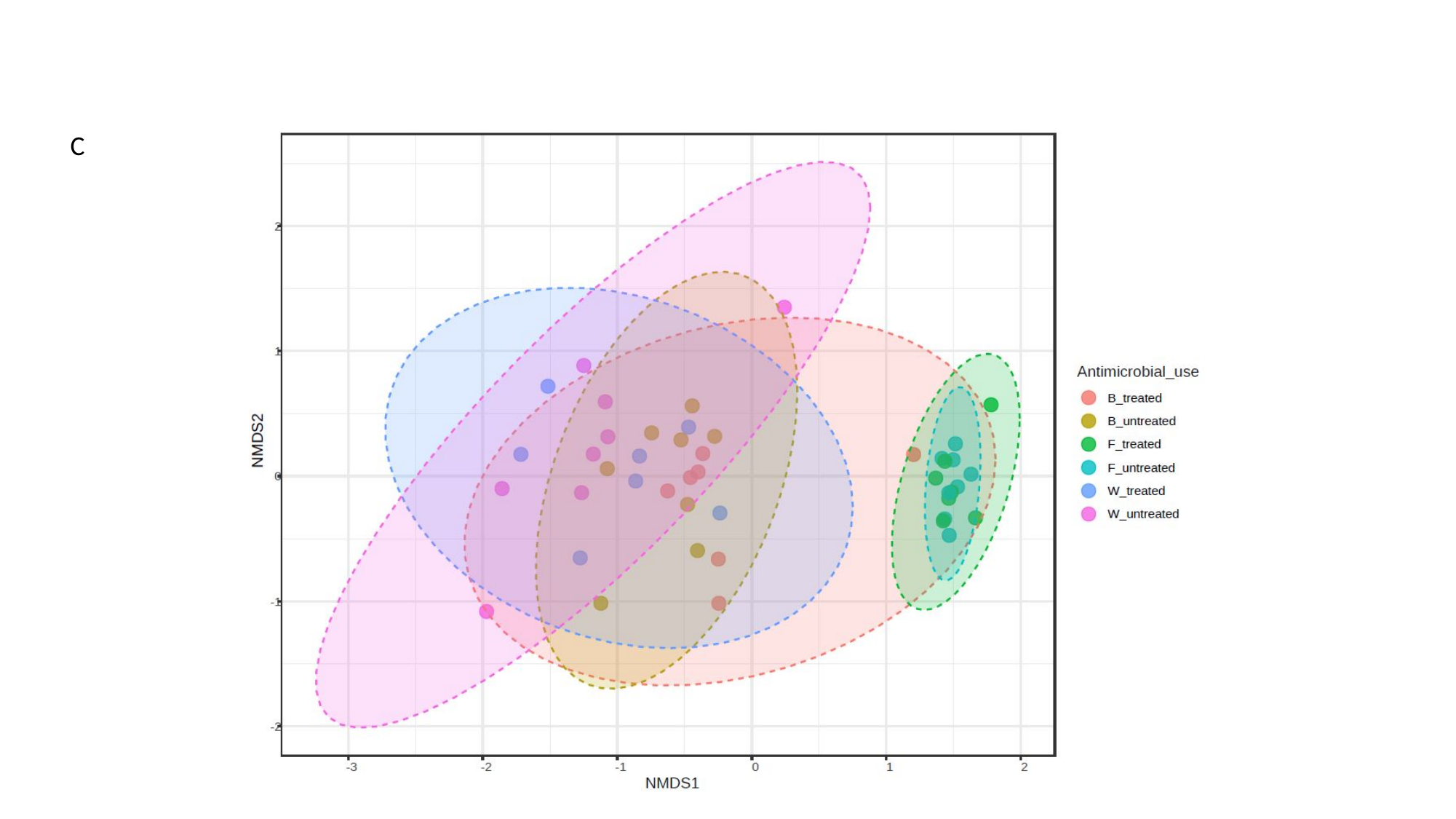

C

## Slide 4
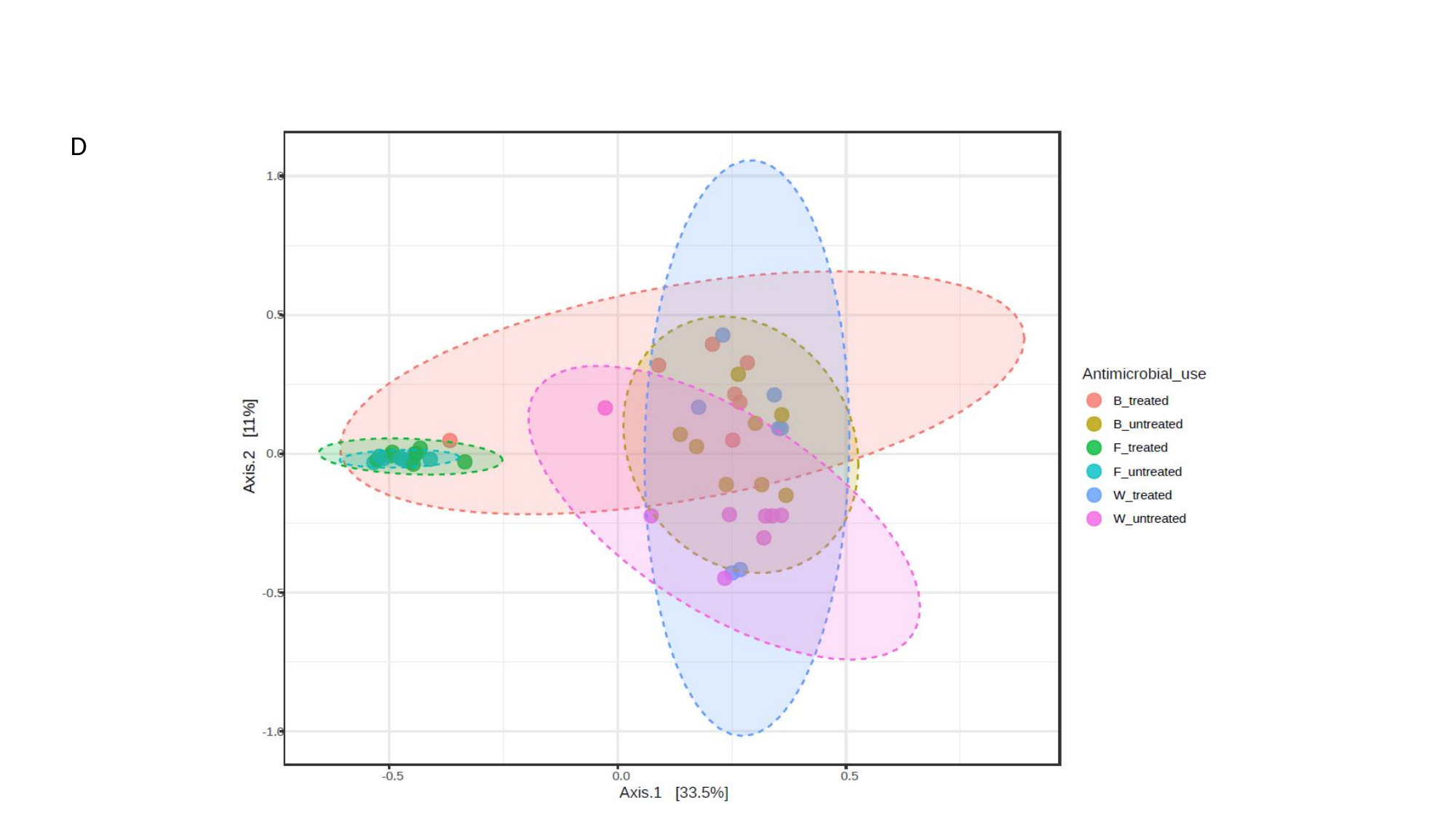

D
