# Supplementary material for: Resistance to medically important antimicrobials in broiler and layer farms in Cameroon and its relation with biosecurity and antimicrobial use
Source: Front Microbiol. 2025 Jan 15;15:1517159. doi: 10.3389/fmicb.2024.1517159 (PMC11774882; doi:10.3389/fmicb.2024.1517159)
Supplement: SUPPLEMENTARY MATERIAL 3 — The list of assays, primers pairs, melting temperatures, and positive controls used in the study. [file Presentation_2.PPTX]

## Slide 1
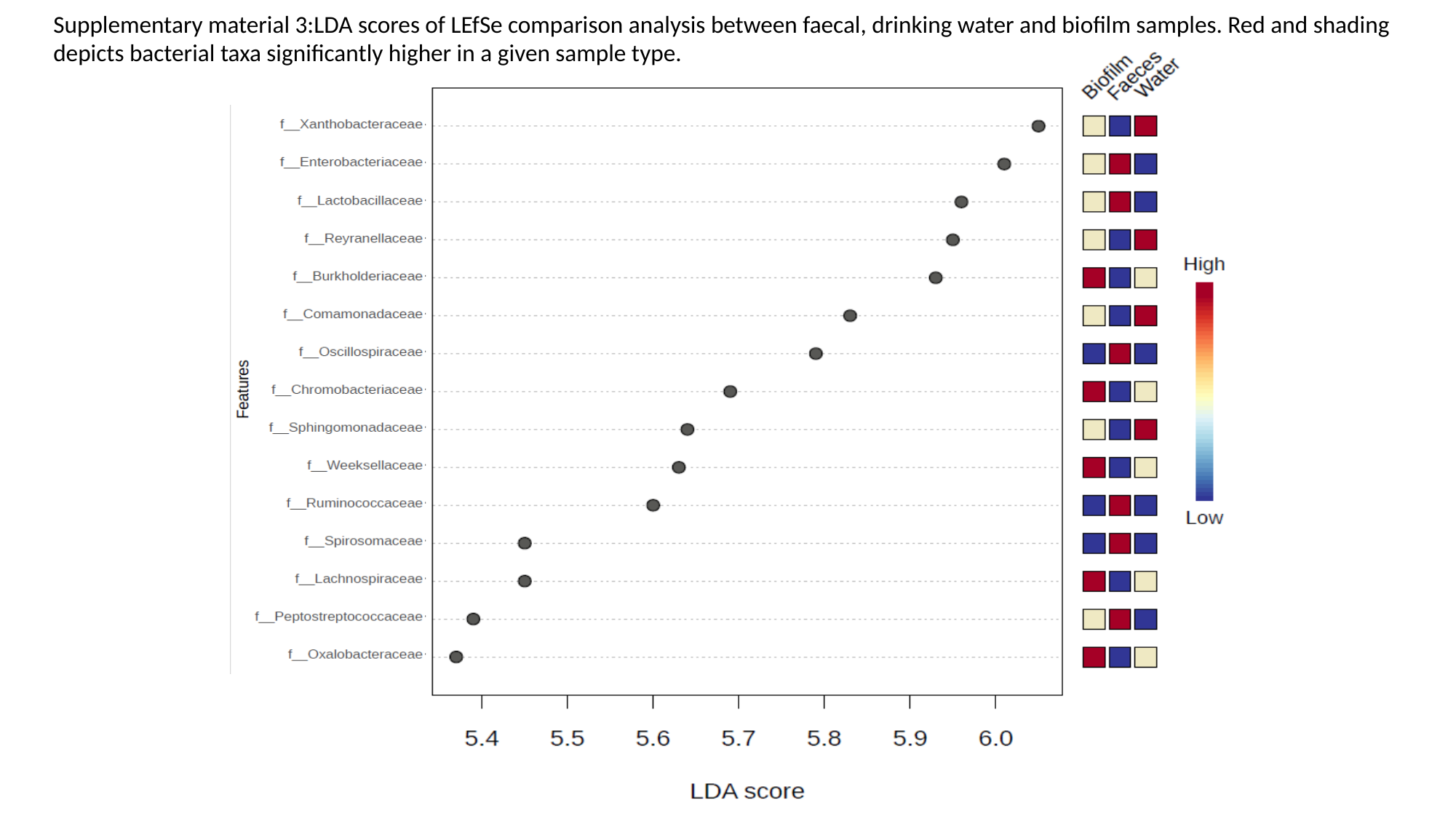

Supplementary material 3:LDA scores of LEfSe comparison analysis between faecal, drinking water and biofilm samples. Red and shading depicts bacterial taxa significantly higher in a given sample type.
