# Supplementary material for: Resistance to medically important antimicrobials in broiler and layer farms in Cameroon and its relation with biosecurity and antimicrobial use
Source: Front Microbiol. 2025 Jan 15;15:1517159. doi: 10.3389/fmicb.2024.1517159 (PMC11774882; doi:10.3389/fmicb.2024.1517159)
Supplement: SUPPLEMENTARY MATERIAL 4 — α-and β-Diversity according to sample type and antimicrobial use. [file Presentation_3.PPTX]

## Slide 1
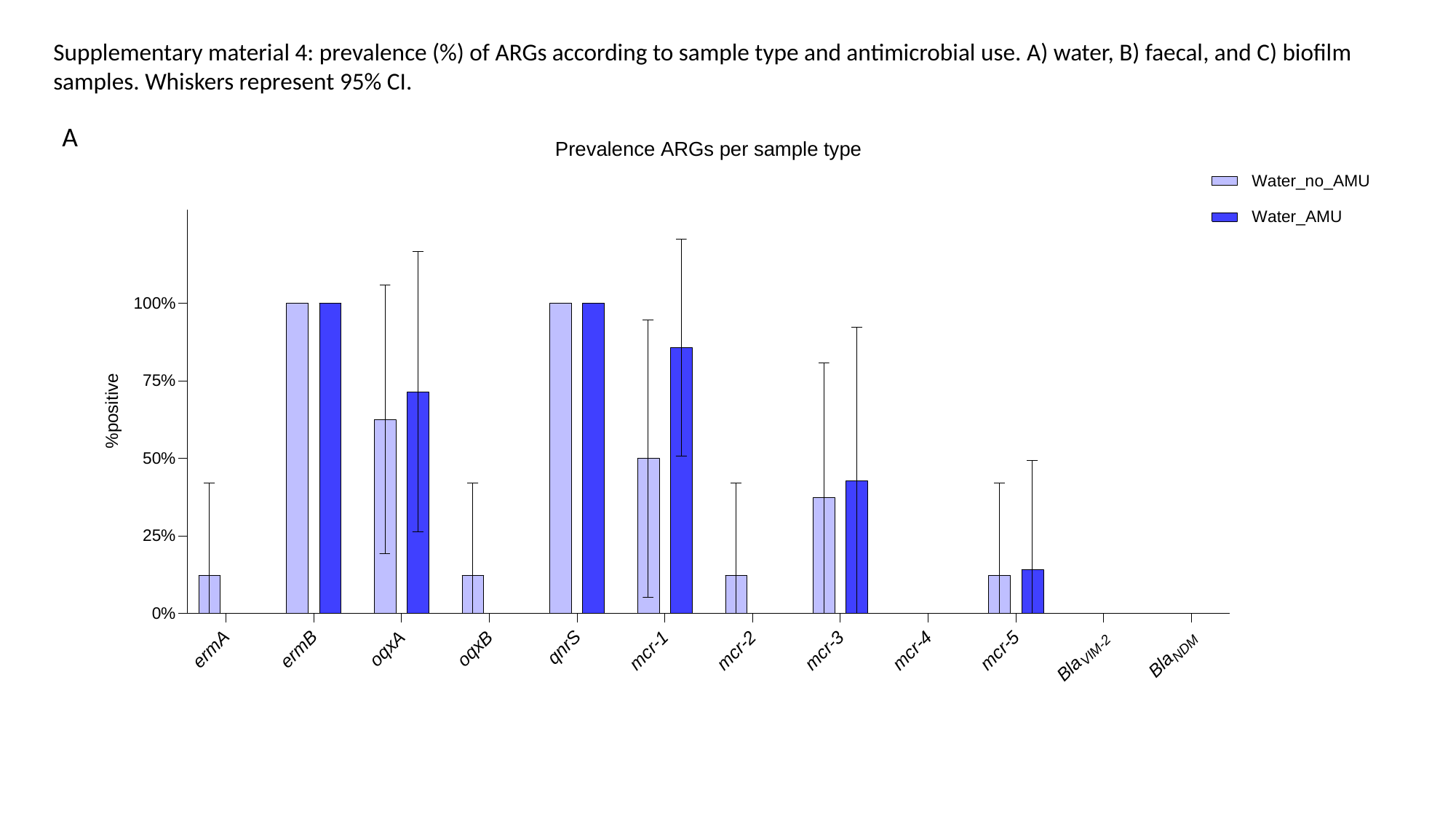

Supplementary material 4: prevalence (%) of ARGs according to sample type and antimicrobial use. A) water, B) faecal, and C) biofilm samples. Whiskers represent 95% CI.
A

## Slide 2
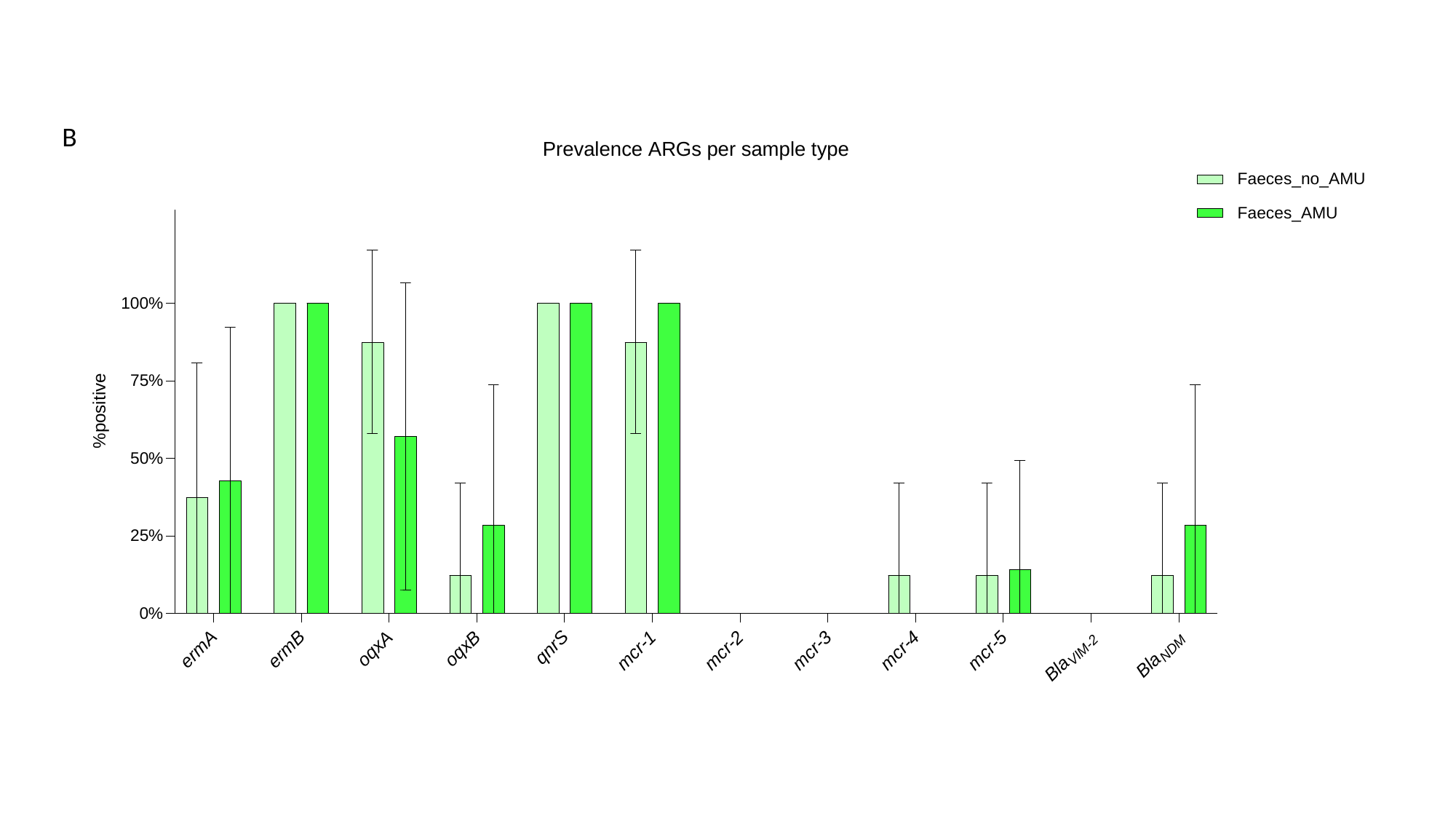

B

## Slide 3
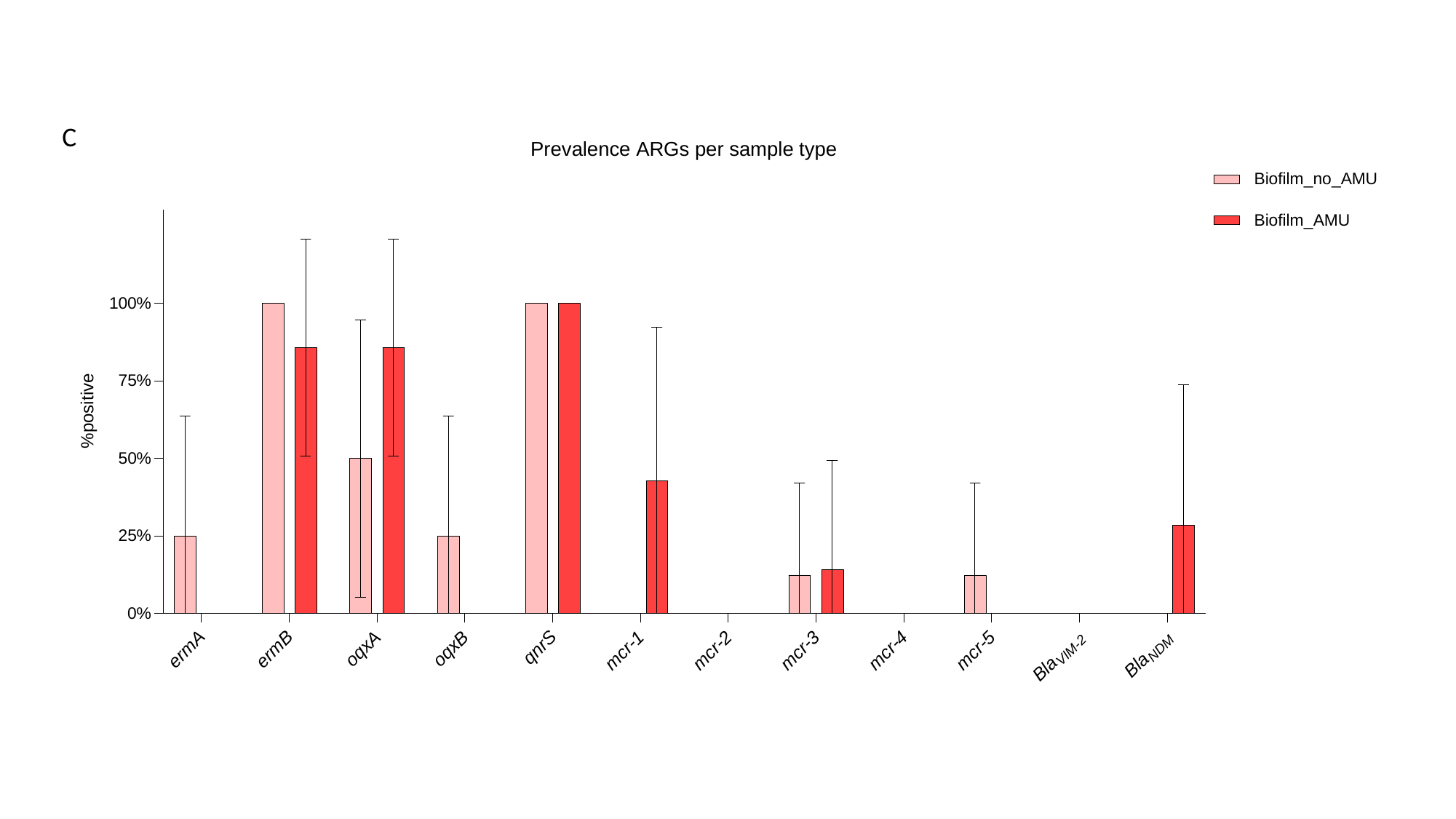

C
